# How to promote the sustainable development of virtual reality technology for training in construction filed: A tripartite evolutionary game analysis

**DOI:** 10.1371/journal.pone.0290957

**Published:** 2023-09-01

**Authors:** Chunyan Shi, Xinyue Miao, Hui Liu, Yang Han, Yafei Wang, Weijun Gao, Gen Liu, Siwen Li, Yingzi Lin, Xindong Wei, Tongyu Xu

**Affiliations:** 1 School of Municipal and Environmental Engineering, Jilin Jianzhu University, Changchun, Jilin province, China; 2 Faculty of Environmental Engineering, The University of Kitakyushu, Kitakyushu, Fukuoka, Japan; 3 School of Emergency Science and Engineering, Jilin Jianzhu University, Changchun, Jilin province, China; 4 Irnovation Institute for Sustainable Maritime Architecture Research and Technology, Qingdao University of Technology, Qingdao, Shandong province, China; 5 School of Environment, Northeast Normal University, Changchun, Jilin province, China; Xuzhou University of Technology, CHINA

## Abstract

In recent years, virtual reality training technology (VRTT) has been considered by many scholars as a new training method instead of traditional training (TT) to reduce unsafe behaviors ascribed to construction workers (CWs) and corporate accident rates. However, in this process, a conflict of interest arises among the government, construction enterprises (CEs), and CWs. Therefore, this study introduces a quantitative research method, the three-party evolutionary game and creatively combining them with the product life cycle (PLC) to solve this problem by analyzing the equilibrium and evolutionarily stable strategies of the system. Finally, collaborative players’ decision-making behaviors and their sensitivity to critical factors are examined. This paper will illustrate these in each stage through numerical simulations. The results of the study indicate that the government plays a dominant role in the VRTT introduction stage. When the government gives CEs appropriate subsidies, CEs will eventually realize the importance of VRTT for CWs. Then the government will gradually reduce the amount of the subsidies in this process. In addition, we also find that the continually high cost will lead to negative policies by the government, which requires the active cooperation and attitude change from CEs and CWs. Ultimately, the government, CEs and CWs adopt the best strategy in the evolutionary process to facilitate the promotion, application and sustainability of VRTT in the construction industry.

## Introduction

The construction industry is one of the most high-risk industries around the world [1]. Chinese government has issued a series of laws and regulations titled Construction Law, Production Safety Law, and Regulation for Production Safety of Construction Projects to regulate procedures and standards relating to construction safety management. Despite improvement of construction safety management are made in the construction industry over the past decades, a large number of accidents continue to occur. According to a report released by China’s Ministry of Housing and Urban-Rural Development, there were 3,622 accidents and 4,198 deaths on construction sites across the country between 2017 and 2021. Over the past five years, the growth rates of accidents and deaths have generally shown a downward trend, decreasing 2.5% and 5.7%, respectively[2]. The annual number of accidents and fatalities has not decreased significantly, which suggests that safety remains a major concern in China’s construction industry.

Heinrich believes that unsafe behaviors of people and unsafe states of objects are the direct causes of accidents, 88% of which were caused by unsafe behaviors of people and 10% by unsafe states of objects. Similarly, risky behaviors by workers contributes to construction accidents. Though China has made significant improvement in creating a safer building environment, little is known about eliminating unsafe behaviors among CWs[3]. Consequently, regulating the performance of managers and the behaviors of workers will dramatically reduce the risk and probability of accidents on the construction site[4].

To reduce the unsafe behaviors of the CWs, security experts and academics further explore innovative ways to solve this issue. Training is one of the results of safety research. Regular but effective training may refresh the understanding of current employees and provide new workers with crucial safety knowledge[5]. There are studies that show the importance of digital transformation and smart technologies in sustainable development [6]. And the VRTT fits into this idea which provides a new medium for CWs to reduce the occurrence of risks from the source. In contrast to TT, VRTT is a more immersive and realistic alternative, where participants are unable to stay focus after the first hour. Those trained in traditional courses risk higher after training, while those trained in VR lower their assessment of risk. This result is consistent with the result from Burke and some other researchers, that the most engaging methods of safety training are the most effective in reducing negative outcomes such as accidents[7]. VRTT has been being strategically introduced into the construction industry. And many pilot studies and applications have been conducted in AEC. With the progress of VRTT and the expansion of the VR equipment market, a growing number of CWs have the opportunity to experience VR technology [8].

However, when VRTT is used as a new training method, the cost of CEs has greatly increased due to the purchase of equipment, talent introduction, equipment maintenance, etc., which leads to some small and medium-sized CEs no longer considering the introduction of VR technology to train CWs. There is a wide array of stakeholders of CEs. Eleven groups of stakeholders have been identified, namely, employees, customers, shareholders, creditors, suppliers and partners, environment and resources agencies, local communities, government, competitors, and NGOs[9]. In this paper, we mainly illustrate the stakeholders of VRTT implementation: the government as the regulators, CEs as the implementers, and CWs as the main participants. In the case of information asymmetry, if you want to find an optimal strategy, game modeling is the best choice [10]. At the same time, this is a dynamic process, these three parts observe, imitate, learn and change strategies from each other during the interaction process [11]. So, in this study, the dynamic evolution of the game is revealed in this research to shed light on the decision-making processes of the government, CEs, and CWs.

In this study, firstly, the influencing elements affect the behavioral strategies of each participant, and the interaction mechanism between them will be described, followed by numerical simulation to get the evolutionary trajectory of the system and the influence of key parameters. Finally, an innovative combination of PLC theory is used to propose mechanism to promote the sustainable development of VRTT in the construction field for different development situations. This research will allow each participant to maximize the benefit of the process and promote the mechanism of sustainable development of VRTT in the construction field. At the same time, this paper enriches the relevant literature on the dissemination of construction safety training, which is important for promoting the promotion, application and sustainable development of VRTT in the construction field.

**Table:** 

**Nomenclature**	
**Parameters**	**Variables**
**N** the income that the government receives	**x** the probability of the government choosing positive strategies
**S** subsidies of the government to CEs when adopting VRTT	**y** the probability of CEs adopting VRTT
**P** penalties to CEs when adopting TT	**z** the probability of CWs receiving VRTT
**C**_**G**_ cost when the government interferes (publicity, etc.)	
**R**_**G1**_ revenue of the government adopting TT	
**R**_**G2**_ additional revenue of the government adopting VRTT	**Acronyms**
**C**_**E1**_ cost for CEs adopting TT	**VR** virtual reality
**C**_**E2**_ additional cost for CEs adopting VRTT	**EGT** evolutionary game theory
**R**_**E1**_ revenue of CEs when adopting TT	**ESS** evolutionary stable strategy
**R**_**E2**_ additional revenue of CEs when adopting VRTT	**VRTT** virtual reality technology for training
**C**_**W1**_ cost for CWs when adopting TT	**TT** traditional training
**C**_**W2**_ additional cost for CWs when adopting VRTT	**CE(s)** construction enterprise(s)
**R**_**W1**_ revenue when adopting TT	**CW(s)** construction worker(s)
**R**_**W2**_ revenue when adopting VRTT	**PLC** product life cycle
**α** the coefficient of the subsidies for CWs adopting VRTT when the government interferes	**Ep** expected payoff **EP** equilibrium point

## Literature review

### VRTT in construction safety training

VRTT has been successfully applied to a variety of vocational training and has achieved remarkable results in different industries [12–14], such as manufacturing [15–17], aviation [18], robotic surgery [19, 20] and mining [21]. Like Accenture, they compared the video training with more immersive VRTT. The study shows that those workers who used VRTT showed a 12% accuracy rate and a 17% increase in the speed at which they completed tasks. When UPS added VRTT to its 11 driver training centers around the world for five days, the drivers’ memorization of the training increased by 75%. JetBlue, United Rentals and Fidelity are also using VR to aid employee training. Tyson is seeing its casualty rate dropped by 20% a year through basic VR-based safety awareness training [22]. These examples fully demonstrate that VRTT has the advantages of improving productivity and reducing risks.

In the construction field, safety training has been proven to improve CWs’ safety awareness and change their unsafe behaviors, which is an important tool to prevent construction site accidents and ensure safe construction [23, 24]. However, most construction sites in China use TT training for CWs, such as videos, handouts, hands-on training, lectures, text-based education, or 2D visual guides, which require CWs to remain focus during training, otherwise they will not be able to change the safety goals of the construction site [25]. It has been shown that as training methods become more engaging, CWs show more attention and less exposure to injury [26]. Researchers have found that introducing VRTT into construction safety training can achieve this goal by not only effectively maintaining CWs’ attention and concentration, but also by better identifying the hazards present on real-world construction sites and increasing CWs’ engagement [27, 28]. CWs will actively explore and enhance the experience, resulting in better acceptance of knowledge from safety training, and then successfully apply those skills and knowledge in real-world settings as effectively or more effectively than TT [29, 30]. Meanwhile, VRTT has been shown to be adequate and acceptable in simulating construction sites and promoting learning and knowledge retention [28, 31, 32]. In addition to promoting learning and thus improving the safety behaviors of CWs, VR-based safety training has also been shown to enhance safety motivation and self-efficacy in identifying safety hazards. There are perceptible effects on both short and long term safety behaviors (knowledge retention) [33].

In China, VRTT has made very little progress in the construction sector. This is because VRTT is in the introduction stage in the construction field, and CEs have high investment and operation costs at this time, and the behavior change of CWs is something that needs to be demonstrated by long-term practice. Therefore, to change this situation, the government should take a series of measures to encourage CEs to adopt VRTT.

### Applicability of the evolutionary game theory

In this study, the government wants more CEs to adopt VRTT to reduce unsafe behaviors of CWs and accidents in the construction [34]. However, CEs are often reluctant to adopt VRTT. On the one hand, the input cost is too high and there are no more benefit in sight in the short term to offset these additional costs. On the other hand, it is difficult for CWs to adopt VRTT without incentives. Therefore, the government must provide incentives to CEs [35]. However, this decision requires a corresponding contribution from the government, so there will be hesitation on the part of the government to implement an active policy. In this case, a conflict of interest between the government, CEs, and CWs has emerged.

In this development process, the interests of the parties are constantly in conflict, and their interactions are similar to a game [36, 37]. Game theory provides a mathematical basis for this by examining how participants develop their choice strategies in the real world [38–40], and predict the final outcome based on assumptions about their behaviors [41]. In recent years, EGT has been used to study the strategic interactions of different stakeholders. For example, Feng et al. (2017) used EGT to study the behavioral evolutionary trend of precast producers [42]. Jing et al. (2020) developed an evolutionary game model of developers’ speculative behaviors in seeking private benefit during PRB advancement [43]. EGT is the most influential economic analysis tool and decision theory for understanding and analyzing conflict and cooperation in the decision-making process [44]. Unlike classical game theory, which assumes that participants are rational, EGT argues that people’s decision-making behaviors in real-world situations achieve dynamic equilibrium through continuous learning, imitation, and trial-and-error, emphasizing dynamic stability [45–47]. It is considered to be an effective method for the dynamic change of multi-intelligentsia strategies in finite rational long-term repeated games. In this process studied in this paper, it is difficult for participants to make completely rational decisions [48]. They need to modify their strategies according to the feedback from their peers [49, 50]. Therefore, EGT is more suitable for describing more complex situation in this study. The four phases of EGT are model description, analysis of dynamic evolution process, verification of stability of strategy combinations and system simulation [51]. This study attempts to construct this model and address the issue of how to understand the dynamic iterative process of the government, business and enterprise in strategic interaction, and to provide a more comprehensive view of the government policy transforming and implementing paths in the context of VRTT.

### Product life cycle theory

**Fig 1:**
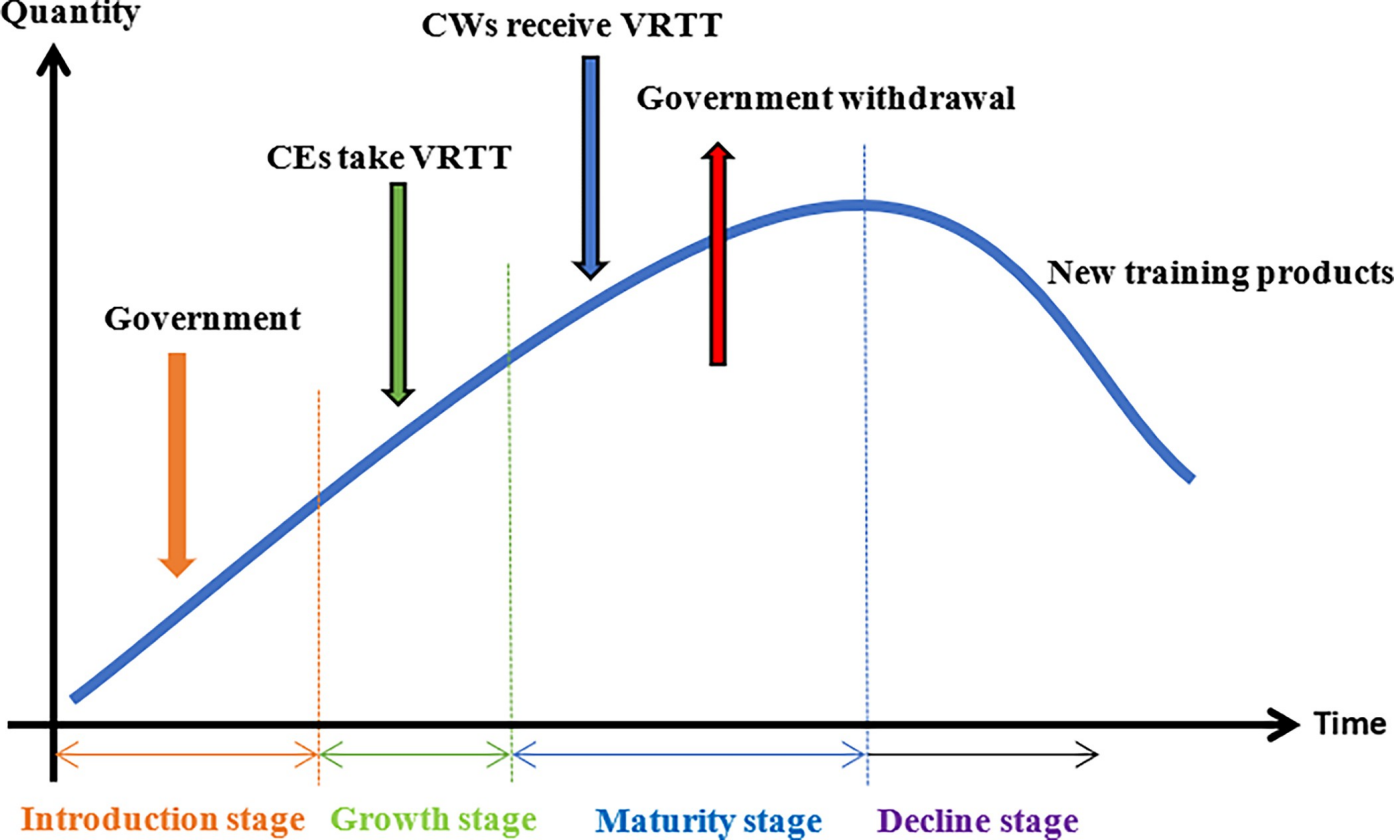
The PLC process of VRTT.

As a new training product, VRTT conforms to the PLC theory. Raymond Vernon believes that the product life refers to the marketing life in the market [52]. The life of a product is the same as that of a human being, which has to go through such cycles as formation, growth, maturity and decline. In the case of a product, it goes through a phase of development, introduction, growth, maturity and decline. VRTT is in the introduction stage of the PLC, which is put into the market test stage. At this time, CEs is in the wait-and-see stage and will not actually buy the product. The government will invest a large amount of money to promote the product. More and more CEs have found that VRTT have good impact on CWs. Thus, CEs choose to adopt VRTT. After the growth stage, VRTT is in a stable stage, and the number of CWs receiving VRTT increased. VRTT enters the mature stage of PLC, which is also the peak period of product benefit. At this time, the government will gradually withdraw from the market, but it will not affect the strategy selection of CEs and CWs. With the development of science and technology, the products will be updated and eventually better training products will replace VRTT, which is in the declining period of PLC. The PLC process of VRTT is as follows (Fig 1).

### Analysis of the interactions of key stakeholders

#### Government

In China, the number of CEs exceeds 1.2 million. Under the circumstance that the country vigorously promotes production safety, the accident rate of CEs has not been significantly reduced, the accident record indicates that most of the architectural accidents are related to human failure, thus unsafe behaviors of CWs are the main cause of the accident [53].The government is actively looking for solutions to change this situation, and VRTT abroad can improve the safe behaviors of CWs. Therefore, the government will implement a number of incentive policies such as subsidies, publicity and so on. However, the government plays as a regulator in this process. The government will not blindly increase subsidies, but always plays a positive guiding role. When CEs choose to adopt VRTT or CWs accept VRTT, the government will withdraw from participation.

#### CEs

In the early days when VRTT entered the construction field, CEs did not realize that adopting VRTT could reduce unsafe behaviors of CWs and enterprise accident rate. Under the gradual subsidies of the government, some CEs choose to adopt VRTT, and achieve good training effects. If the number of the enterprise accidents decrease gradually, it will certify the stable strategy of CEs that tend to adopt VRTT. At the same time, economic benefit is the ultimate pursuit of any enterprise [54]. Even if the government withdraws from the market after VRTT products enter the mature stage, CEs, which see the long-term safety benefit of VRTT, will actively adopt VRTT.

#### CWs

CEs will give out a part of subsidies to encourage more CWs to accept VRTT. Some CWs prefer to receive TT in a short period of time, due to their limited education level, cognition and limited acceptance of new things. However, CWs will gradually realize that VRTT improves operation the capability and their own safety benefit in the long term. When CEs gradually adopt VRTT and increase the subsidy coefficient for CWs receiving VRTT under the government’s strategy, CWs will gradually shift from TT to VRTT.

**Fig 2:**
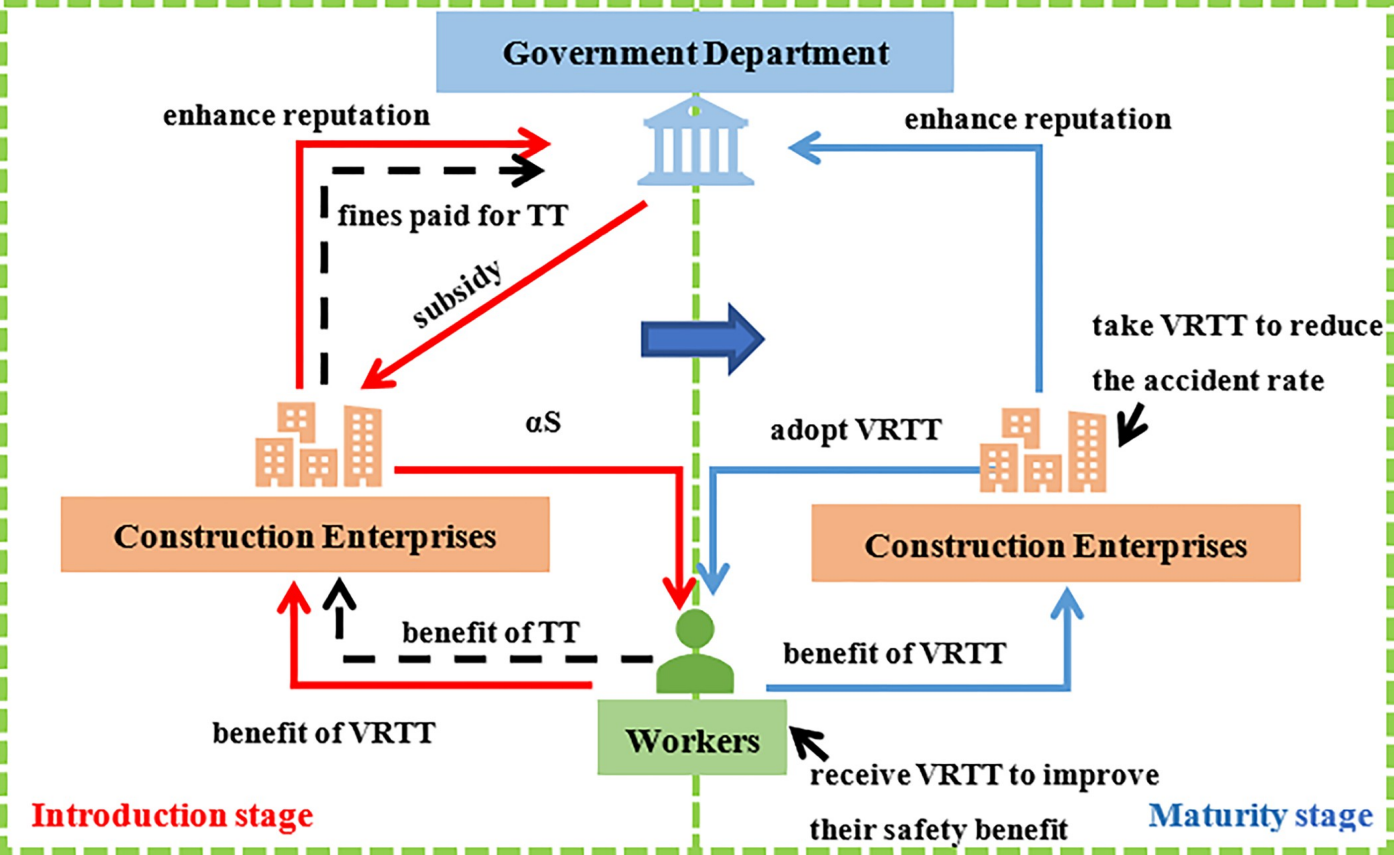
The relationship among the Government, CEs and CWs.

The relationship among the government, CEs and CWs (Fig 2) are shown as follows.

## Tripartite evolutionary game modeling

First, a hypothesis of EGT model involving government, CEs and CWs is presented. Then, a tripartite EGT model is established, and its equilibrium solution is obtained by setting the replicated dynamic equation. And then you get the Jacobian of the system. Finally, the stable strategy of the system is analyzed based on Lyapunov stability theory [55].

### Assumptions and payoff matrix

#### Assumption1

The EGT model includes three stakeholders: government, CEs and CWs (received both VRTT and TT). They are capable of independent decision-making, bound rationality, and seek to maximize their own interests. Furthermore, they can adapt and learn to shifts in their surroundings, as well as modify their tactics during VRTT.

#### Assumption 2

Assuming that the probability of the government adopts a positive strategy to encourage CEs to adopt VRTT is *x*(0≤*x*≤1), the probability of the government adopts negative strategies (no subsidies, no publicity, etc.) is 1−*x*; the probability of CEs choosing VRTT is *y*(0≤*y*≤1), the probability of CEs choosing TT is 1−*y*. The probability of CWs choosing VRTT is *z*(0≤*z*≤1), the probability of CWs choosing TT is 1−*z*.

#### Assumption 3 (government)

When the government adopts a positive strategy, publicity, guidance and experience of VRTT will generate cost C_G_, improve the public’s awareness of VRTT and gain reputation N. At this time, if CEs adopt TT, the government will punish them P, but TT reduces the unsafe behaviors of CWs, and enables the healthy development of CEs, and bring revenue for the government R_G1_; if CEs adopt VRTT, the government will provide subsidies S. Compared with TT, VRTT can reduce unsafe behaviors of CWs to a greater extent, thus greatly reducing the accident rate of construction site and bringing additional revenue to the government R_G2_.

#### Assumption 4 (CEs)

When CEs use TT to train CWs, they will cost of organizing training for security personnel, purchasing books and security-related television materials C_E1_ and TT enhances safety awareness of CWs and reduces unsafe behaviors of workers, thus making CEs gain profits R_E1_. If the CEs adopt VRTT, the purchase of VR equipment, the increase of related technicians and equipment maintenance will generate additional costs C_E2_. However, with the further reduction of unsafe behaviors of CWs, the occurrence of accidents in CEs will be reduced, and get increased revenue R_E2_.

#### Assumption 5 (CWs)

When CWs choose to accept TT, the training fee is C_W1_, while the training reduces unsafe behaviors, and their own safety benefit are R_W1_; when CWs choose to accept VRTT, the additional training fee is C_W2_, and CEs reward them with αS (0<*α*<1), CWs further reduce unsafe behaviors, and the additional safety benefit is R_W2_.

Based on the above assumptions, the payoff matrix of the tripartite game model is established and shown in Table 1.

**Table 1:** Payoff matrix of the tripartite EGT model.

Players		Workers	
		Accept VRT (z)	Accept TT (1-z)
**positive strategy** (**x**)	**VRT(y)**	**I**_**1**_ **= *N*−*S*−*C***_***G***_**+*R***_***G*1**_**+*R***_***G*2**_	**I**_**2**_ **= *N*−*S*−*C***_***G***_**+*R***_***G*1**_**+*R***_***G*2**_
		**II**_**1**_ **= (1−*α*)*S*−(*C***_***E*1**_**+*C***_***E*2**_**)+(*R***_***E*1**_**+*R***_***E*2**_**)**	**II**_**2**_ **= *S*−(*C***_***E*1**_**+*C***_***E*2**_**)+(*R***_***E*1**_**+*R***_***E*2**_**)**
		**III**_**1**_ **= *αS*−(*C***_***W*1**_**+*C***_***W*2**_**)+(*R***_***W*1**_**+*R***_***W*2**_**)**	III_2_ = 0
	**TT** (**1-y**)	I_3_ = *N*+*P*−*C*_*G*_+*R*_*G*1_	I_4_ = *N*+*P*−*C*_*G*_+*R*_*G*1_
		II_3_ = −*P*−*C*_*E*1_+*R*_*E*1_	II_4_ = −*P*−*C*_*E*1_+*R*_*E*1_
		III_3_ = 0	III_4_ = −*C*_*W*1_+*R*_*W*1_
**negative strategy** (**1-x**)	**VRT(y)**	**I**_**5**_ **= *R***_***G*1**_**+*R***_***G*2**_	**I**_**6**_ **= *R***_***G*1**_**+*R***_***G*2**_
		**II**_**5**_ **= −(*C***_***E*1**_**+*C***_***E*2**_**)+(*R***_***E*1**_**+*R***_***E*2**_**)**	**II**_**6**_ **= −(*C***_***E*1**_**+*C***_***E*2**_**)+(*R***_***E*1**_**+*R***_***E*2**_**)**
		**III**_**5**_ **= −(*C***_***W*1**_**+*C***_***W*2**_**)+(*R***_***W*1**_**+*R***_***W*2**_**)**	III_6_ = 0
	**TT** (**1-y**)	I_7_ = *R*_*G*1_	I_8_ = *R*_*G*1_
		II_7_ = −*C*_*E*1_+*R*_*E*1_	II_8_ = −*C*_*E*1_+*R*_*E*1_
		III_7_ = 0	III_8_ = −*C*_*W*1_+*R*_*W*1_

### Stakeholders’ replication dynamic equation

Let *U*_*x*1_ represents the Ep of the government if adopting a positive strategy, and *U*_*x*2_ represents the expected payoff (Ep) of the government if adopting a negative strategy. *U*_*x*_ represents the average Ep of the government. *U*_*x*1_, *U*_*x*2_ and *U*_*x*_ can be expressed as:

$$U_{x1}=yzI_{1}+y(1-z)I_{2}+(1-y)zI_{3}+(1-y)(1-z)I_{4}=N+P-C_{G}+R_{G1}+y(R_{G2}-P-S)$$ (1)

$$U_{x2}=yzI_{5}+y(1-z)I_{6}+(1-y)zI_{7}+(1-y)(1-z)I_{8}=R_{G1}+yR_{G2}$$ (2)

$$U_{x}=xU_{x1}+(1-x)U_{x2}=x(N+P-Py-Sy-C_{G})+R_{G1}+yR_{G2}$$ (3)

According to Eqs (1)–(3), we obtain the replicator dynamics equation of the government can be written as:

$$F(x)=x(1-x)(U_{x1}-U_{x2})=x(1-x)[N+P-(P+S)y-C_{G}]$$ (4)

*U*_*y*1_ represents the Ep of the CEs if they choose VRTT, and *U*_*y*2_ represents the Ep of the CEs if they choose TT. *U*_*y*_ represents the average Ep of the CEs. *U*_*y*1_, *U*_*y*2_ and *U*_*y*_ can be expressed as:

$$U_{y1}=xzII_{1}+x(1-z)II_{2}+(1-x)zII_{5}+(1-x)(1-z)II_{6}=x(S-Sz\alpha )-C_{E1}-C_{E2}+R_{E1}+R_{E2}$$ (5)

$$U_{y2}=xzII_{3}+x(1-z)II_{4}+(1-x)zII_{7}+(1-x)(1-z)II_{8}=-Px-C_{E1}+R_{E1}$$ (6)

$$U_{y}=yU_{y1}+(1-y)U_{y2}=-Px-C_{E1}+R_{E1}+y(Px+Sx(1-z\alpha )-C_{E2}+R_{E2})$$ (7)

According to Eqs (5)–(7), we obtain the replicator dynamics equation of the CEs can be written as:

$$F(y)=y(1-y)(U_{y1}-U_{y2})=y(1-y)[x(P+S-Sz\alpha )-C_{E2}+R_{E2}]$$ (8)

In the same way, *U*_*z*1_ represents the Ep of the CWs if they choose to accept VRTT, and *U*_*z*2_ represents the CWs if they choose to accept TT. *U*_*z*_ represents the average Ep. *U*_*z*1_, *U*_*z*2_ and *U*_*z*_ can be expressed as:

$$U_{z1}=xyIII_{1}+x(1-y)III_{3}+(1-x)yIII_{5}+(1-x)(1-y)III_{7}=y(Sx\alpha -C_{W1}-C_{W2}+R_{W1}+R_{W2})$$ (9)

$$U_{z2}=xyIII_{2}+x(1-y)III_{4}+(1-x)yIII_{6}+(1-x)(1-y)III_{8}=(y-1)(C_{W1}-R_{W1})$$ (10)

$$\begin{array}{l} Uz=zUz1+(1-z)Uz2=-C_{W1}+zC_{W1}+R_{W1}-zR_{W1}+\\ y[C_{W1}-R_{W1}+z(Sx\alpha -2C_{W1}-C_{W2}+2R_{W1}+R_{W2})] \end{array}$$ (11)

According to Eqs (9)–(11), we obtain the replicator dynamics equation of the CWs can be written as:

$$F(z)=z(1-z)(U_{z1}-U_{z2})=z(1-z)[C_{W1}-R_{W1}+y(Sx\alpha -2C_{W1}-C_{W2}+2R_{W1}+R_{W2})]$$ (12)

The replicator dynamic equations of the government, CEs and CWs constitute a three-dimensional dynamic system, as shown in Eq (13):

$$\{\begin{array}{c} F(x)=x(1-x)[N+P-(P+S)y-C_{G}]\\ F(y)=y(1-y)[x(P+S-Sz\alpha )-C_{E2}+R_{E2}]\\ F(z)=z(1-z)[C_{W1}-R_{W1}+y(Sx\alpha -2C_{W1}-C_{W2}+2R_{W1}+R_{W2})] \end{array}$$ (13)

### Analysis of game evolution path

**Fig 3:**
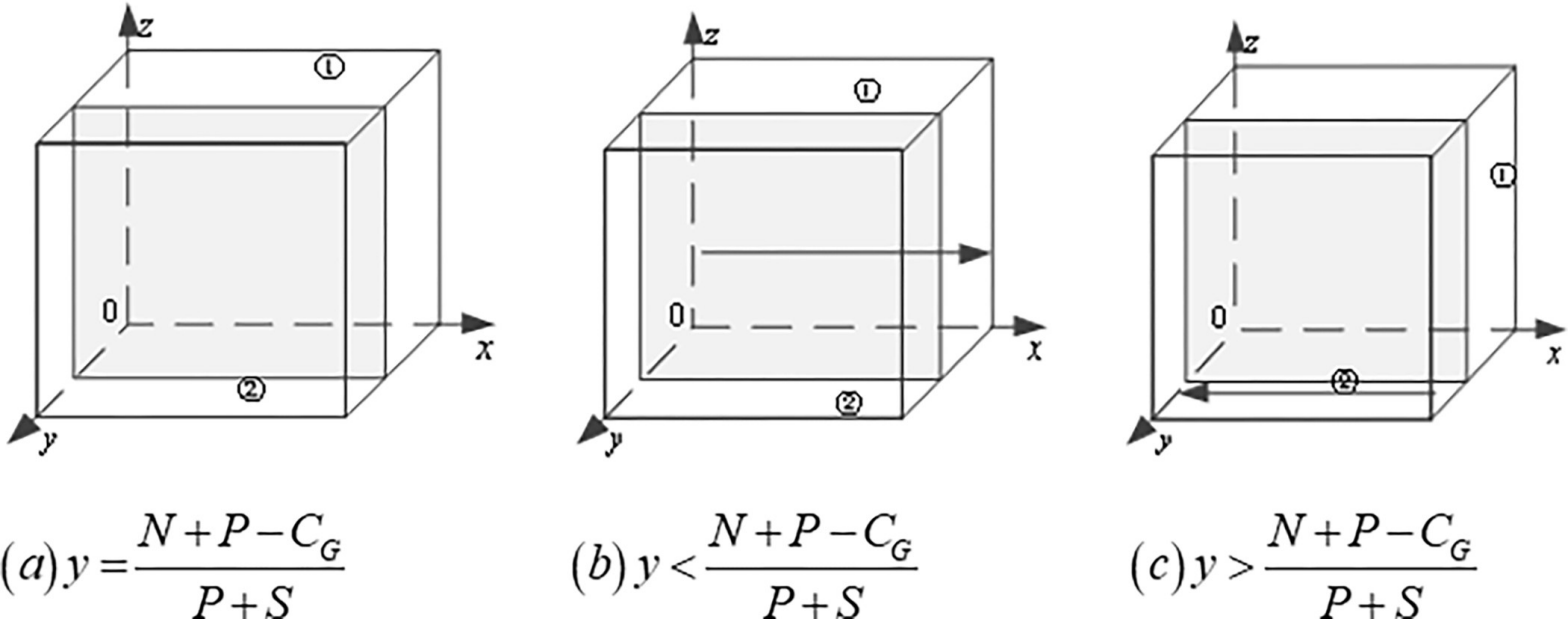
Dynamic evolution path of the government’s strategies.

Firstly, the progressive stability analysis of the government is carried out: Based on (4), when $y=\frac{N+P-C_{G}}{P+S}$, the replicator dynamic equation *F*(*x*) = 0, which means that the game system is always in a stable state at this time, and the government’s strategy selection ratio x does not change with time. When $y\neq \frac{N+P-C_{G}}{P+S}$, we obtain two stable points, i.e., *x* = 0 and *x* = 1, according to the EGT, if $F^{\prime }(x)=(1-2x)[N+P-(P+S)y-C_{G}]<0$, then the stable point represented by x is valid. The evolutionary stability depends on the morphology of the interface $N+P-(P+S)y-C_{G}=0$, when $y<\frac{N+P-C_{G}}{P+S}$, $F^{\prime }(x){|}_{x=0}>0$, $F^{\prime }(x){|}_{x=1}<0$, in this case, *x* = 1 is the evolutionary stable point, the government choose positive strategy. When $y>\frac{N+P-C_{G}}{P+S}$, $F^{\prime }(x){|}_{x=0}<0$, $F^{\prime }(x){|}_{x=1}>0$, in this case, *x* = 0 is the evolutionary stable point, and the government choose the negative strategies, as shown in (Fig 3).

**Fig 4:**
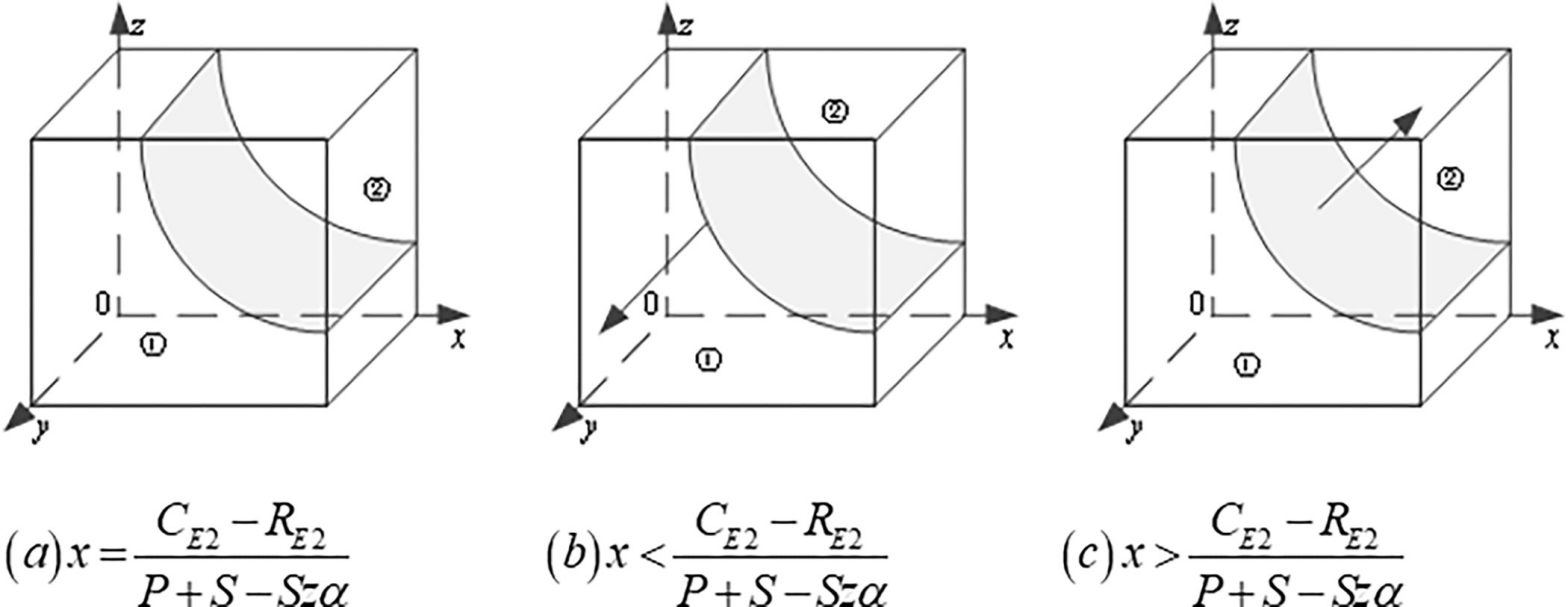
Dynamic evolution path of the CEs’ strategies.

Secondly, the gradual stability analysis of CEs: Based on (8), when $x=\frac{C_{E2}-R_{E2}}{P+S-Sz\alpha }$, the replicator dynamic equation is *F*(*y*)≡0, which means that the game system is always in a stable state. At this time, CEs strategy selection ratio y does not change with time. When $x\neq \frac{C_{E2}-R_{E2}}{P+S-Sz\alpha }$, we obtain two stable points, i.e., *y* = 0 and *y* = 1, according to the EGT, if $F^{\prime }(y)=(1-2y)[x(P+S-Sz\alpha )-C_{E2}+R_{E2}]<0$, then the stable point represented by y is valid. The evolutionary stability depends on the morphology of the interface *x*(*P+S−Szα*)−*C*_*E*2_+*R*_*E*2_ = 0, when $x<\frac{C_{E2}-R_{E2}}{P+S-Sz\alpha }$, $F^{\prime }(y){|}_{y=0}<0$, $F^{\prime }(y){|}_{y=1}>0$, in this case, *y* = 0 is the evolutionary stable point, and CEs choose to adopt TT to train CWs. When $x>\frac{C_{E2}-R_{E2}}{P+S-Sz\alpha }$, $F^{\prime }(y){|}_{y=0}>0$, $F^{\prime }(y){|}_{y=1}<0$, in this case, *y* = 1 is the evolutionary stable point, and CEs choose to adopt VR to train CWs (Fig 4).

**Fig 5:**
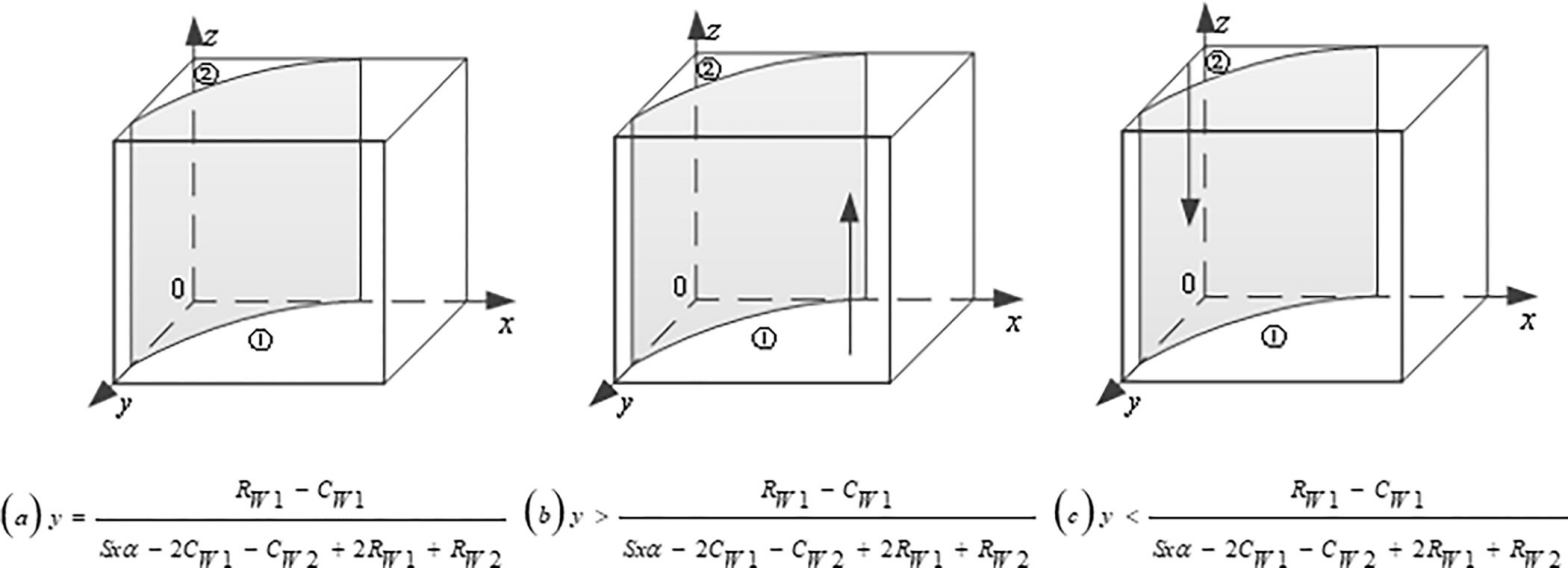
Dynamic evolution path of the CWs strategies.

Finally, the gradual stability analysis of CWs: Based on (12), when $y=\frac{R_{W1}-C_{W1}}{Sx\alpha -2C_{W1}-C_{W2}+2R_{W1}+R_{W2}}$, the replicator dynamic equation is *F*(*z*)≡0, which means that the game system is always in a stable state. At this time, CWs strategy selection ratio z does not change with time. When $y\neq \frac{R_{W1}-C_{W1}}{Sx\alpha -2C_{W1}-C_{W2}+2R_{W1}+R_{W2}}$, we obtain two stable points, i.e., *z* = 0 and *z* = 1, according to the EGT, if $F^{\prime }(z)=(1-2z)[C_{W1}-R_{W1}+y(Sx\alpha -2C_{W1}-C_{W2}+2R_{W1}+R_{W2})]<0$, then the stable point represented by z is valid. The evolutionary stability depends on the morphology of the interface. $C_{W1}-R_{W1}+y(Sx\alpha -2C_{W1}-C_{W2}+2R_{W1}+R_{W2})=0$, when $y>\frac{R_{W1}-C_{W1}}{Sx\alpha -2C_{W1}-C_{W2}+2R_{W1}+R_{W2}}$, $F^{\prime }(z){|}_{z=0}>0$, $F^{\prime }(z){|}_{z=1}<0$, in this case, *z* = 1 is the evolutionary stable point, and CWs choose to accept VRTT. When $y<\frac{R_{W1}-C_{W1}}{Sx\alpha -2C_{W1}-C_{W2}+2R_{W1}+R_{W2}}$, $F^{\prime }(z){|}_{z=0}<0$, $F^{\prime }(z){|}_{z=1}>0$, in this case, *z* = 0 is the evolutionary stable point, and CWs still choose to receive TT, as shown in (Fig 5).

### Solve the equilibrium point (EP) of evolutionary game

When *F*(*x*) = 0, *F*(*y*) = 0 and *F*(*z*) = 0, according to Eq (13), E_1_(0,0,0), E_2_(1,0,0), E_3_(0,1,0), E_4_(0,0,1), E_5_(1,1,0), E_6_(1,0,1), E_7_(0,1,1) and E_8_(1,1,1) of the system can be determined. There are four mixed-strategies EPs that conform to Eq (14). If e*(x*, y*, z*) does not belong to (0,1), e* should be rejected.

$$\{\begin{array}{c} N+P-(P+S)y-C_{G}=0\\ x(P+S-Sz\alpha )-C_{E2}+R_{E2}=0\\ C_{W1}-R_{W1}+y(Sx\alpha -2C_{W1}-C_{W2}+2R_{W1}+R_{W2})=0 \end{array}$$ (14)

To identify the evolutionary stable strategy, the stability of these EPs must be analyzed. The Jacobian matrix is applicable to evaluate the evolution equilibrium stability [56]. The Jacobian matrix is given by (15):

$$\begin{array}{l} J=\left[\begin{array}{ccc} \frac{\partial F(x)}{\partial x} & \frac{\partial F(x)}{\partial y} & \frac{\partial F(x)}{\partial z}\\ \frac{\partial F(y)}{\partial x} & \frac{\partial F(y)}{\partial y} & \frac{\partial F(y)}{\partial z}\\ \frac{\partial F(z)}{\partial x} & \frac{\partial F(z)}{\partial y} & \frac{\partial F(z)}{\partial z} \end{array}\right]\\ =\left[\begin{array}{ccc} \left(1-2x)[N+P-(P+S)y-C_{G}\right] & x(1-x)(-P-S) & 0\\ y(1-y)(P+S-z\alpha S) & \left(1-2y)[R_{E2}-C_{E2}+x(P+S-zS\alpha )\right] & y(1-y)(-S\alpha x)\\ z(1-z)(yS\alpha ) & z(1-z)(Sx\alpha -2C_{W1}-C_{W2}+2R_{W1}+R_{W2}) & \left(1-2z)[C_{W1}-R_{W1}+y(Sx\alpha -2C_{W1}-C_{W2}+2R_{W1}+R_{W2})\right] \end{array}\right] \end{array}$$ (15)

The EP we found may not be the system’s ESS, and it can only be asymptotically stable when the pure strategy Nash equilibrium is met [55, 57]. We only examine the asymptotic stability of 8 pure strategy EPs in this study since e* is a mixed strategy Nash equilibrium. To obtain the eigenvalues of each EP shown in Table 2, we substitute them into Eq (15).

Using Lyapunov’s notion of stability [58]:

1. If all eigenvalues *λ*<0, then the corresponding EP is stable, and the point is ESS.
2. If all eigenvalues *λ*>0, then the corresponding EP is unstable.
3. If there are both *λ*>0 and *λ*<0, then the corresponding EP is unstable and is also a saddle point.

Finally, the asymptotic stability of these EPs can be verified by analyzing the eigenvalues of the Jacobian matrix of the system [56].

**Table 2:** Eigenvalues and stability conditions for each EP.

Points	Eigenvalues			Conditions of ESS
	λ_1_	λ_2_	λ_3_	
***E***_**1**_ **(0,0,0)**	*N*+*P*−*C*_*G*_	−*C*_*E*2_+*R*_*E*2_	*C*_*W*1_−*R*_*W*1_	*λ*_1_<0,*λ*_2_<0,*λ*_3_<0
***E***_**2**_ **(1,0,0)**	−*N*−*P*+*C*_*G*_	*P*+*S*−*C*_*E*2_+*R*_*E*2_	*C*_*W*1_−*R*_*W*1_	*λ*_1_<0,*λ*_2_<0,*λ*_3_<0
***E***_**3**_ **(0,1,0)**	*N*−*S*−*C*_*G*_	*C*_*E*2_−*R*_*E*2_	−*C*_*W*1_−*C*_*W*2_+*R*_*W*1_+*R*_*W*2_	*λ*_1_<0,*λ*_2_<0,*λ*_3_<0
***E***_**4**_ **(0,0,1)**	*N*+*P*−*C*_*G*_	−*C*_*E*2_+*R*_*E*2_	−*C*_*W*1_+*R*_*W*1_	*λ*_1_<0,*λ*_2_<0,*λ*_3_<0
***E***_**5**_ **(1,1,0)**	−*N*+*S*+*C*_*G*_	−*P*−*S*+*C*_*E*2_−*R*_*E*2_	*αS*−*C*_*W*1_−*C*_*W*2_+*R*_*W*1_+*R*_*W*2_	*λ*_1_<0,*λ*_2_<0,*λ*_3_<0
***E***_**6**_ **(1,0,1)**	−*N*−*P*+*C*_*G*_	*P*+(1−*α*)*S*−*C*_*E*2_+*R*_*E*2_	−*C*_*W*1_+*R*_*W*1_	*λ*_1_<0,*λ*_2_<0,*λ*_3_<0
***E***_**7**_ **(0,1,1)**	*N*−*S*−*C*_*G*_	*C*_*E*2_−*R*_*E*2_	*C*_*W*1_+*C*_*W*2_−*R*_*W*1_−*R*_*W*2_	*λ*_1_<0,*λ*_2_<0,*λ*_3_<0
***E***_**8**_ **(1,1,1)**	−*N*+*S*+*C*_*G*_	−*P*−(1−*α*)*S*+*C*_*E*2_−*R*_*E*2_	−*αS*+*C*_*W*1_+*C*_*W*2_−*R*_*W*1_−*R*_*W*2_	*λ*_1_<0,*λ*_2_<0,*λ*_3_<0

According to the PLC theory, VRTT has entered the market as a new training product. After parts of domestically and overseas CEs adopting VR technology to train CWs, the unsafe behaviors of CWs has been reduced to a large extent, and the accident rate of CEs has also been reduced [53]. Since the product is in the introduction stage, most CEs will not take the initiative to bear the additional cost of VR equipment introduction. At this time, the government chooses the positive incentive strategy to issue a series of preferential policies to CEs, corresponding to the stable point E_2_(1,0,0), which satisfies the inequality: −*N*−*P*+*C*_*G*_<0. Even if TT is adopted by CEs, the income of the government at this time will still be higher than the cost of the active strategy, thus choosing the positive strategy: *P*+*S*−*C*_*E*2_+*R*_*E*2_<0. If so, CEs will choose TT and pay the fine less than the net cost of the situation of adopting VRTT. They will not realize that VRTT reduces the enterprise accident rate and the healthy development of the industry. *C*_*W*1_−*R*_*W*1_<0, in this situation. TT will still be adopted. Since CEs still adopt TT, CWs rarely have access to VRTT. At this time, the benefit of TT for CWs exceeds the costs, so CWs will still receive TT.

With the subsidies of the government, VRTT is in the growth stage of PLC. An increasing number of CEs choose to replace TT with VRTT under the government’s subsidies, which corresponds to the stable point E_5_(1,1,0) and satisfies −*N*+*S*+*C*_*G*_<0. The cost and subsidies provided by the government are less than the benefit brought by the active strategy, so the active strategy will be chosen. −*P*−*S*+*C*_*E*2_−*R*_*E*2_<0, in this case, the penalty paid by CEs for TT is greater than the net cost of using VRTT. To reduce the unsafe behaviors of CWs and improve the safety efficiency of enterprises, CEs use VR technology instead of TT to train CWs, *αS*−*C*_*W*1_−*C*_*W*2_+*R*_*W*1_+*R*_*W*2_<0, in this case, the benefit of VRTT is less than the cost, and CWs still choose to receive TT.

Under the government’s active strategy and CEs’ strategy of replacing TT with VRTT, CWs have more and more opportunities to contact VRTT. Meanwhile, with the passage of time, CWs also realize that VRTT can improve their own safety behaviors. It is in the mature stage of PLC, corresponding to the stable point E_8_(1,1,1), which satisfies −*N*+*S*+*C*_*G*_<0, in this time, the government still adopt the active strategy. −*P*−(1−*α*)*S*+*C*_*E*2_−*R*_*E*2_<0, the penalty paid by CEs for TT is greater than the net cost of VRTT, so VRTT is adopted to train CWs. −*αS*+*C*_*W*1_+*C*_*W*2_−*R*_*W*1_−*R*_*W*2_<0, in this case, the benefit of VRTT is greater than the cost, so CWs choose to accept VRTT.

After the application of VRTT in CEs enters the mature stage, the government will gradually withdraw from the market. At this time, CEs have formed a relatively complete VRTT system. At the same time, after receiving different training methods, CWs realize that VRTT methods will further improve their own safety behaviors, corresponding to the stable point E_7_(0,1,1), which satisfies *N*−*S*−*C*_*G*_<0. Government’s benefit is less than the cost and subsidies paid, so it takes a negative strategy. *C*_*E*2_−*R*_*E*2_<0, in this case, the additional benefit of VRTT for CEs is greater than the additional costs, so VRTT for CWs is adopted. *C*_*W*1_+*C*_*W*2_−*R*_*W*1_−*R*_*W*2_<0, in this case, the benefit of VRTT for CWs is also greater than the costs. Therefore, CWs choose to accept VRTT.

## Numerical simulations and results

In order to more intuitively observe the behaviors evolution of the three-parties in the architecture field at each stage of VRTT, we use MATLAB(R2021a) to conduct numerical simulation. First, illustrate the dynamic evolution trend of the three-parties during the introduction, growth, and maturity (S>0, S = 0) of the VRTT product. In most parts of China, VRTT products are still in the introduction stage, so the government’s strategy is critical to driving the development of the entire construction sector. Therefore, we numerically simulate the changes of government related parameters (i.e. incentive subsidies, governance costs and penalties) to explore their impact on CEs and CWs, providing theoretical reference for the government to promote the development of VRTT in the architectural field to a mature stage.

### Four-stages dynamic evolution results

**Fig 6:**
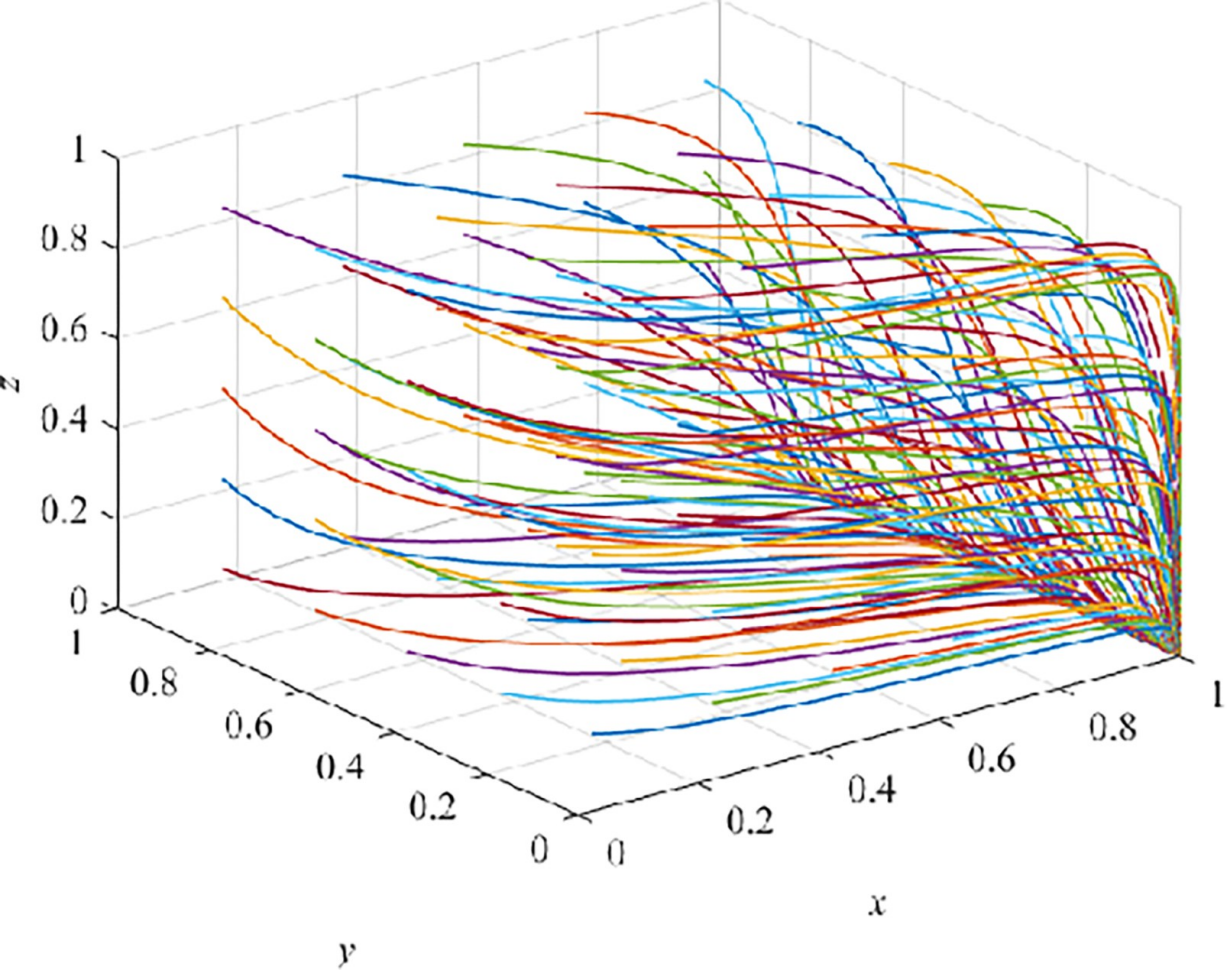
Evolution pathway of introduction stage.

**Fig 7:**
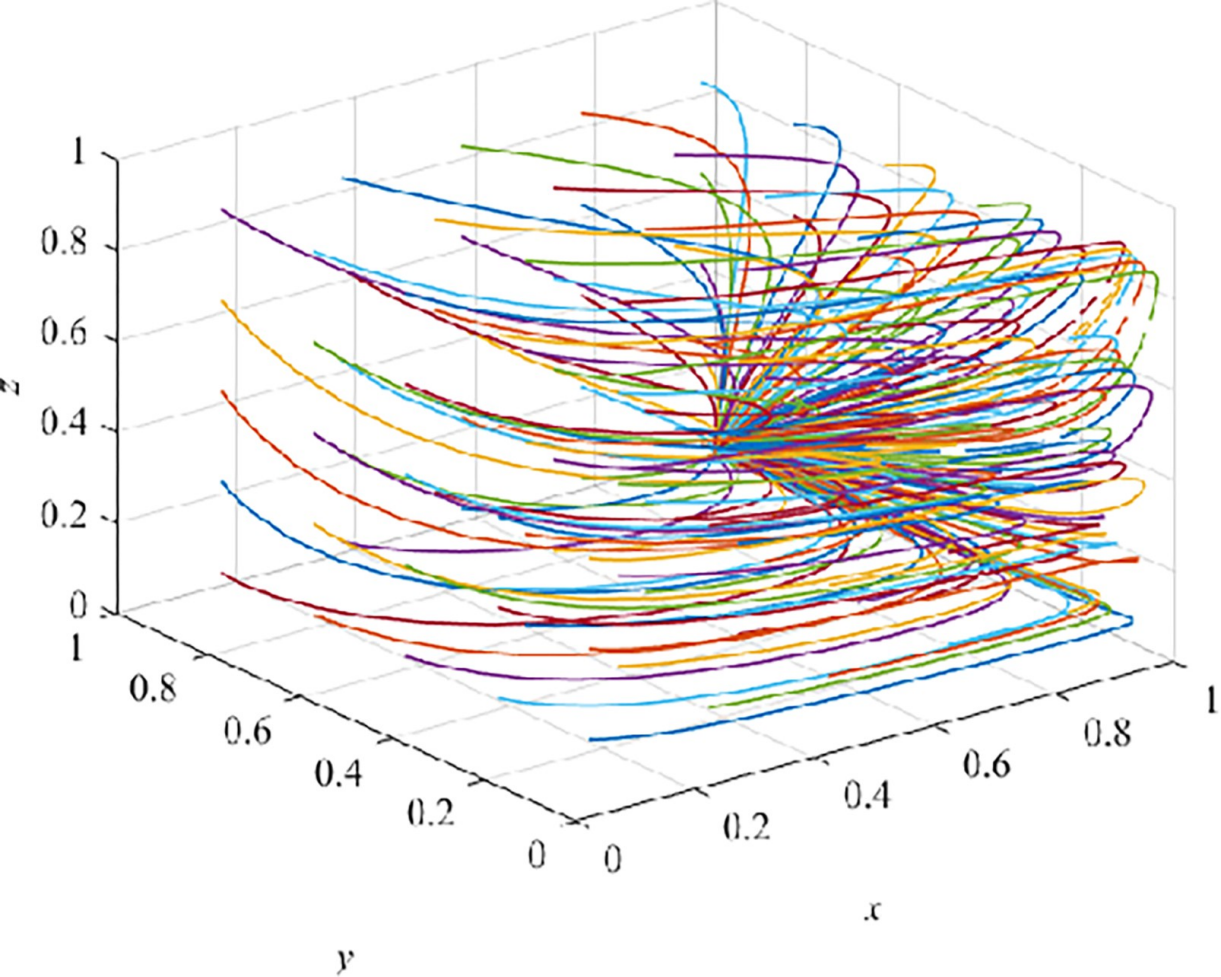
Evolution pathway of growth stage.

**Fig 8:**
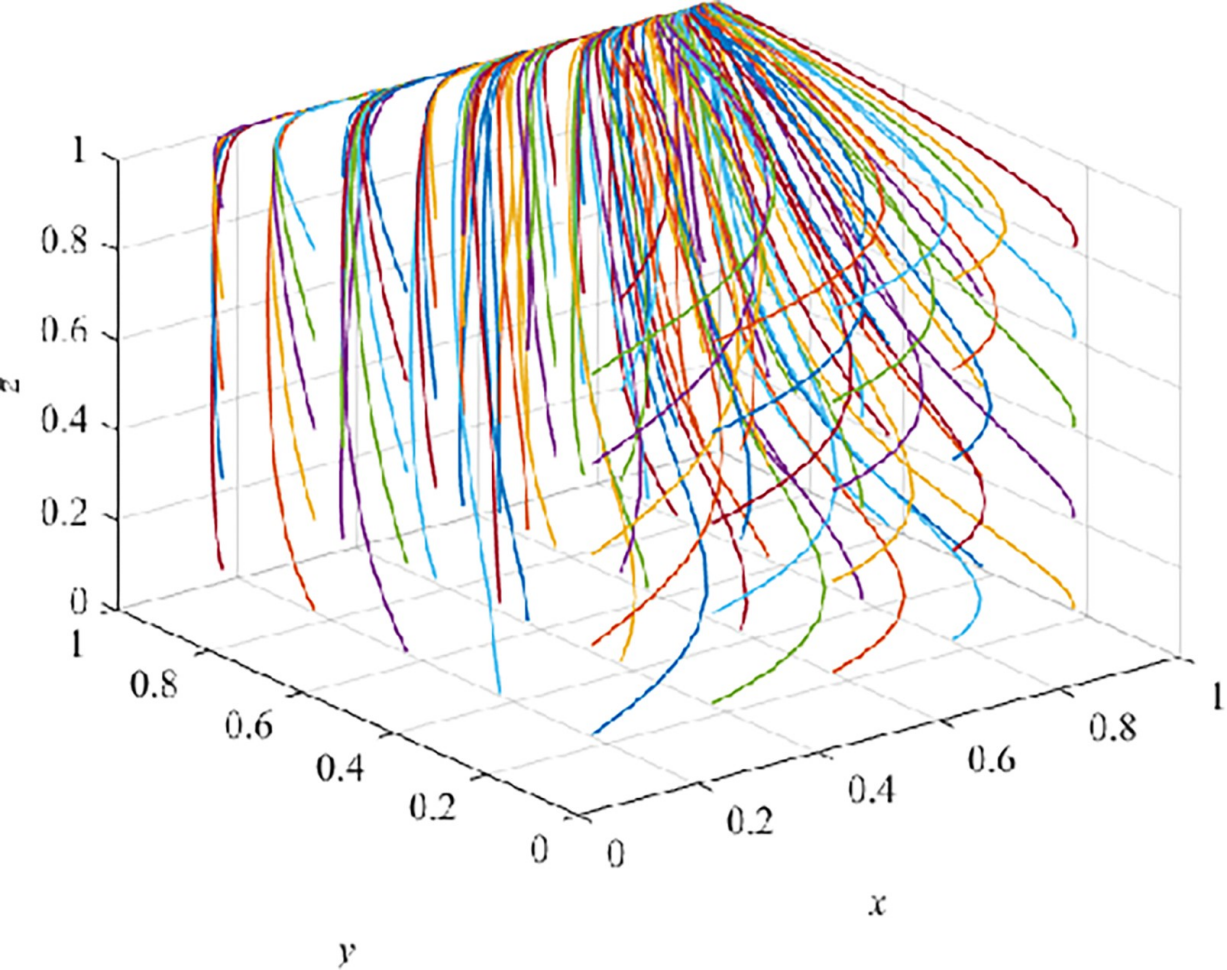
Evolution pathway of maturity stage(S>0).

**Fig 9:**
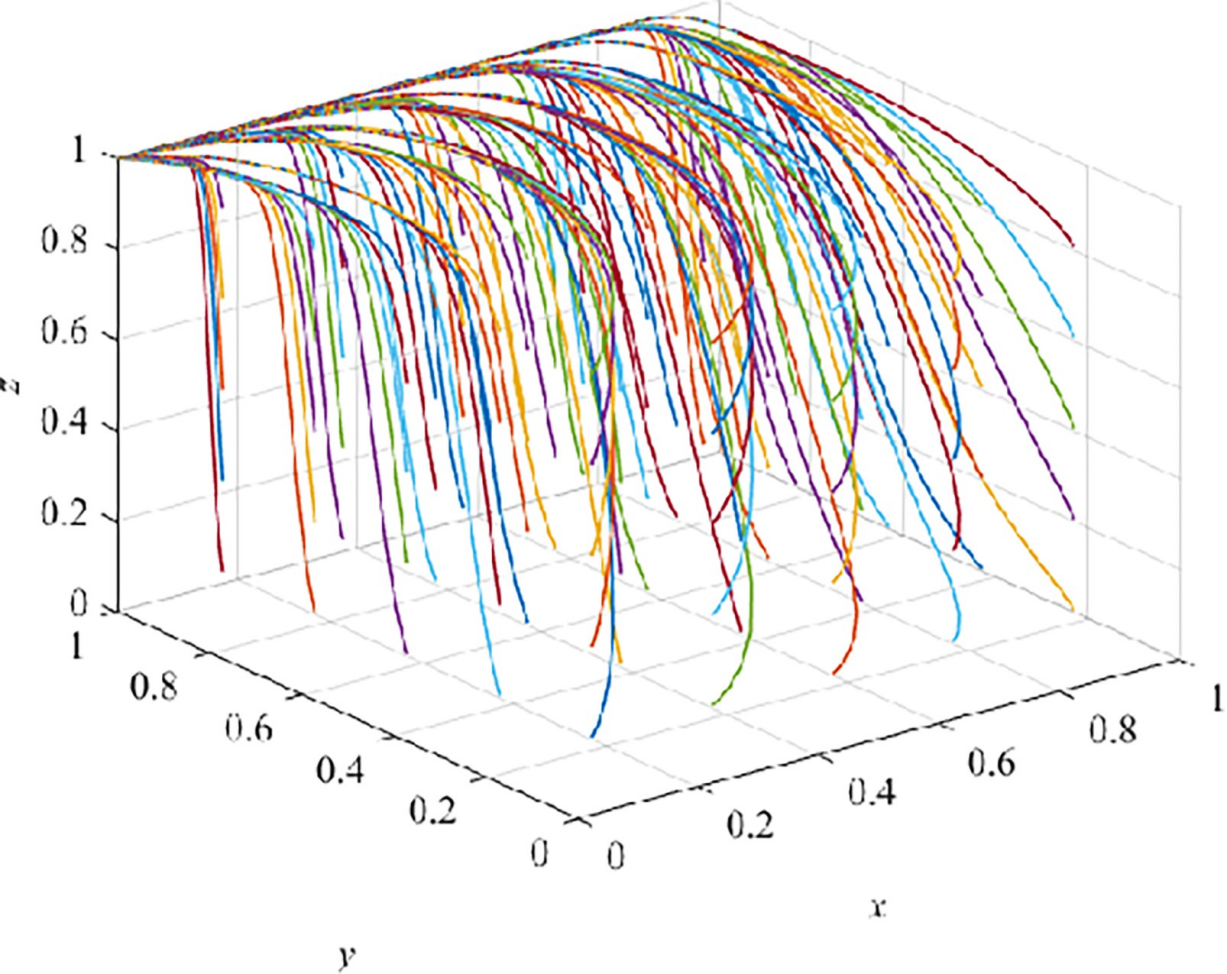
Evolution pathway of maturity stage(S = 0).

As shown in Table 3, each parameter value corresponding to the four stages meets the three stability conditions. We randomly generate 100 groups of tripartite evolutionary game with fixed x, y and z values, as show in (Figs 6–9).

**Table 3:** Values of parameters in the numerical simulation.

Parameters	N	S	P	C_G_	R_G1_	R_G2_	C_E1_	C_E2_	R_E1_	R_E2_	C_W1_	C_W2_	R_W1_	R_W2_	α
**Introduction stage**	18	5	3	5	30	8	10	20	15	11	15	10	16	0	0.2
**Growth stage**	18	10	3	5	30	8	10	10	15	11	15	8	16	0	0.3
**Maturity stage(S>0)**	15	9	3	5	30	8	10	6	15	11	15	6	16	15	0.4
**Maturity stage(S = 0)**	4	0	3	5	30	8	10	10	15	11	15	4	16	20	0.2

### Evolution result of introduction stage

In the introduction stage, after 100 iterations, they all converge to E_2_(1,0,0), and it is also verified that E_2_(1,0,0) is an asymptotically stable EP (Fig 6). At this time, the government adopts a positive strategy, while CEs adopt TT, and CWs receive TT. Under the guarantee constraint, E_3_(1,0,0) is the ESS of the system, which is consistent with the forementioned analyzed result under the guarantee constraint conditions. When VRTT is brought in, the income of CEs and CWs adopting TT exceeds both fines and training costs. At the same time, CEs will refuse to adopt VRTT because of the high cost and too much dependence on TT or lack of social responsibility [27]. In this way, TT is accepted.

In this case, promoting CEs to transform from TT to VRTT has become the primary goal of the government at this stage. However, at the early stage of the use of VRTT, the economic benefit for CEs is not obvious, and the investment in VRTT equipment is large, so it is difficult for CEs to choose VRTT under the pressure of huge funds. In order to ensure the sustainable development of VRTT in the field of architecture, the government should provide CEs with special funds for VRTT and various economic incentives, such as tax incentives, financial subsidies and loan incentives, so as to fully mobilize CEs to change from TT to VRTT. When CEs still adopt TT, which leads to a high accident rate or a large safety accident, the government will impose administrative penalties, fines or orders for rectification on CEs, so as to inhibit CEs’ choice of TT (Fig 6)

### Evolution result of growth stage

In the growth stage, after 100 iterations, they all converge to E_5_(1,1,0), and it is also verified that E_5_(1,1,0) is an asymptotically stable EP (Fig 7). At this time, the government adopts a positive strategy, and CEs adopt VRTT, and CWs receive TT. The system shows that E_5_(1,1,0) is the ESS and enters the product growth stage under the guarantee constraint. The behaviors strategy of CEs will gradually change from TT to VRTT. Because VRTT is a new thing for CWs, moreover, in a short period of time, they cannot realize the advantages of VRTT, such as reducing their unsafe behaviors and improving their safety benefit [59]. Moreover, for CWs, VRTT costs a lot and they get little or no benefit at this stage.

In this case, the government should continue to play a leading role, carry out effective publicity and education, strengthen supervision of CEs, take VR to train CWs, appropriately increase subsidies for CEs, so that CEs can increase the reward for CWs who choose to accept VRTT (α mentioned in the article) to encourage CWs to accept VRTT instead of TT.

### Evolution result of maturity stage (S>0)

In the maturity stage (S>0), after 100 iterations, they all converge to E_8_(1,1,1), and it is also verified that E_8_(1,1,1) is an asymptotically stable EP (Fig 8). At this time, the government adopts a positive strategy, and CEs adopt VRTT, and CWs receive VRTT. Under the guarantee constraint, E_8_(1,1,1) is the ESS of the system at this time, and is also the best advantage of the evolutionary game system. As the product enters the mature stage, the government’s active strategy promotes the development of VRTT in the field of architecture. CEs gain higher income than TT, meanwhile, after receiving two kinds of training in the long-term training process, CWs finally realize the safety benefit brought by VRTT to improve learning efficiency.

In this case, first of all, the government should focus more on VRTT. In particular, the development and upgrading of VRTT should be further perfected to provide basic guarantee for CEs that choose to adopt VRTT. Secondly, the government should improve laws and regulations to restrain some CEs in the adoption of VRTT subsidies to seek private interests of speculation. At the same time, we can also implement a series of effective supervision system, implement mandatory measures, standardize CEs choose to adopt VRTT behaviors. When CEs adopt VRTT and CWs accept it, the application of VRTT in the field of architecture enters a virtuous cycle with the growth of economic benefit, the improvement of safety benefit and the expansion of social influence. However, after the product enters the mature stage, the government will not continue to increase the regulatory cost. In order to confirm this conclusion, we obtain (Fig 9).

### Evolution result of maturity stage(S = 0)

In the maturity stage (S = 0), after 100 iterations, they all converge to E_7_(0,1,1), and it is also verified that E_7_(0,1,1) is an asymptotically stable EP (Fig 9). At this time, the government adopts a negative strategy, CEs adopts VRTT, and CWs receive VRTT. The system shows that E_7_(0,1,1) is the ESS under the guarantee constraint. At this stage, due to the growing social responsibility of CEs and stable comprehensive benefit, the security awareness of CWs is also increasing. Even if the government no longer carries out active strategies such as subsidies, CEs will also choose to take VRTT and CWs will choose to accept VRTT. At this time, the government, as the regulator, can no longer intervene in the market and will withdraw from participation, which refers to economic withdrawal. Government continues to support the media and the Internet, etc., to harness market mechanisms and intangible resources for self-regulation and policy advancement. CEs and CWs will maximize their use of VRTT products in their own development until better training products become available.

### Sensitivity analysis of key parameters on the evolution of tripartite system

**Fig 10:**
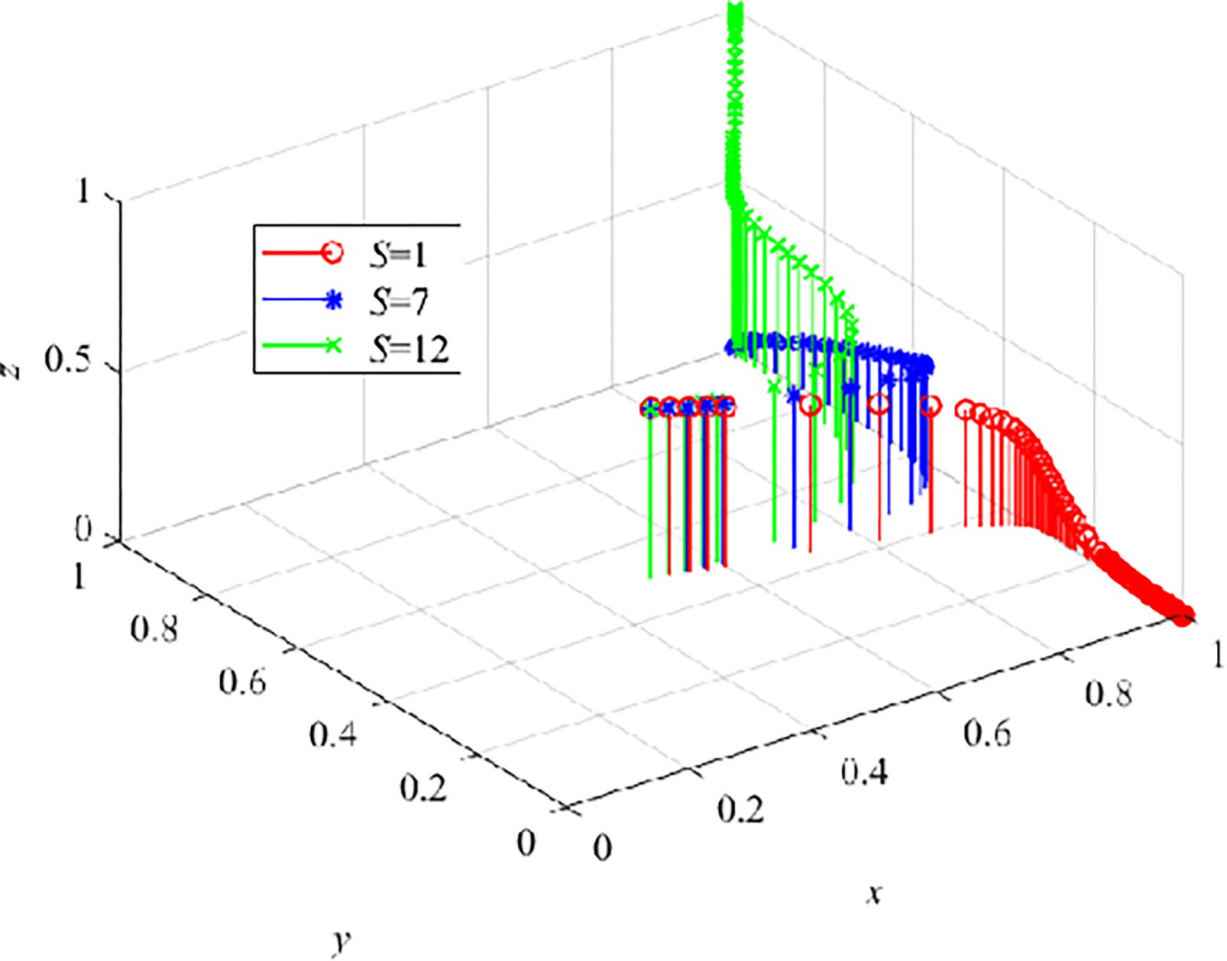
The effect of S on the dynamic evolution trend of the three-parties.

In order to further explore the regulatory role of the government on the development of VRTT, we numerically simulate the influence of penalty P, S, α, and C_G_. First, we assign S as 1, 7, and 12 respectively to conduct numerical simulation of the three-party evolutionary game model. The results are shown in (Fig 10). When S = 1, y and z continuously decrease to 0, indicating that lower subsidies will not make CEs choose to adopt VRTT, and CWs with less contact will not receive such training at this time. CEs and CWs have insufficient understanding of VRTT, the development of VRTT is subject to regulation and direction from the government. As the government appropriately increases subsidies (Fig 10), when S = 7, the stable strategy of CEs tends to adopt VRTT. At this time, it is in the growth stage of the PLC. If the product is to enter the mature stage, it needs to mobilize CWs to accept VRTT. Here, we set S to 12, and the evolutionary game system reaches the best advantage E_8_ (1,1,1).

**Fig 11:**
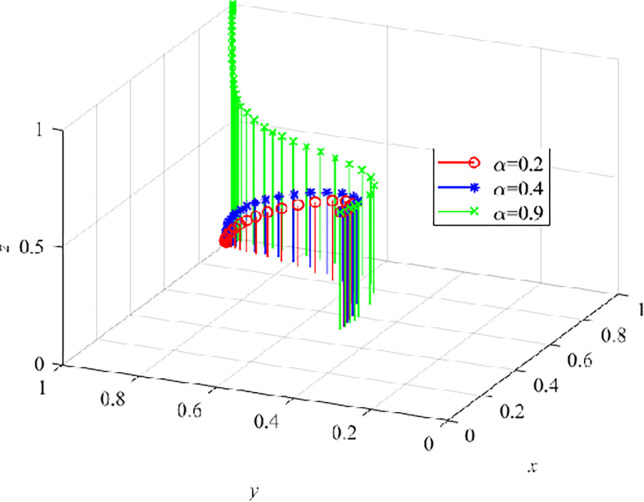
The effect of α on the dynamic evolution trend of the three-parties.

Similarly, the subsidy coefficient α is critical to the degree of VRTT acceptance by CWs. Then, we set α as 0.2, 0.4 and 0.9 respectively to conduct numerical simulation for the three-party evolutionary game model. The results are shown in (Fig 11). When α = 0.2 and α = 0.4, z decreases continuously to 0, indicating that lower subsidy coefficient in the growth stage of VRTT will not make CWs accept VRTT, and with the increase of subsidy coefficient, The results are shown in (Fig 11). When α = 0.9, more and more CWs choose to accept VRTT, which lays a foundation for the government to withdraw from the market. When CWs realize that VRTT improves their safety behaviors, they will accept VRTT under the leadership of CEs, and the system gradually evolve to the optimal stable point.

**Fig 12:**
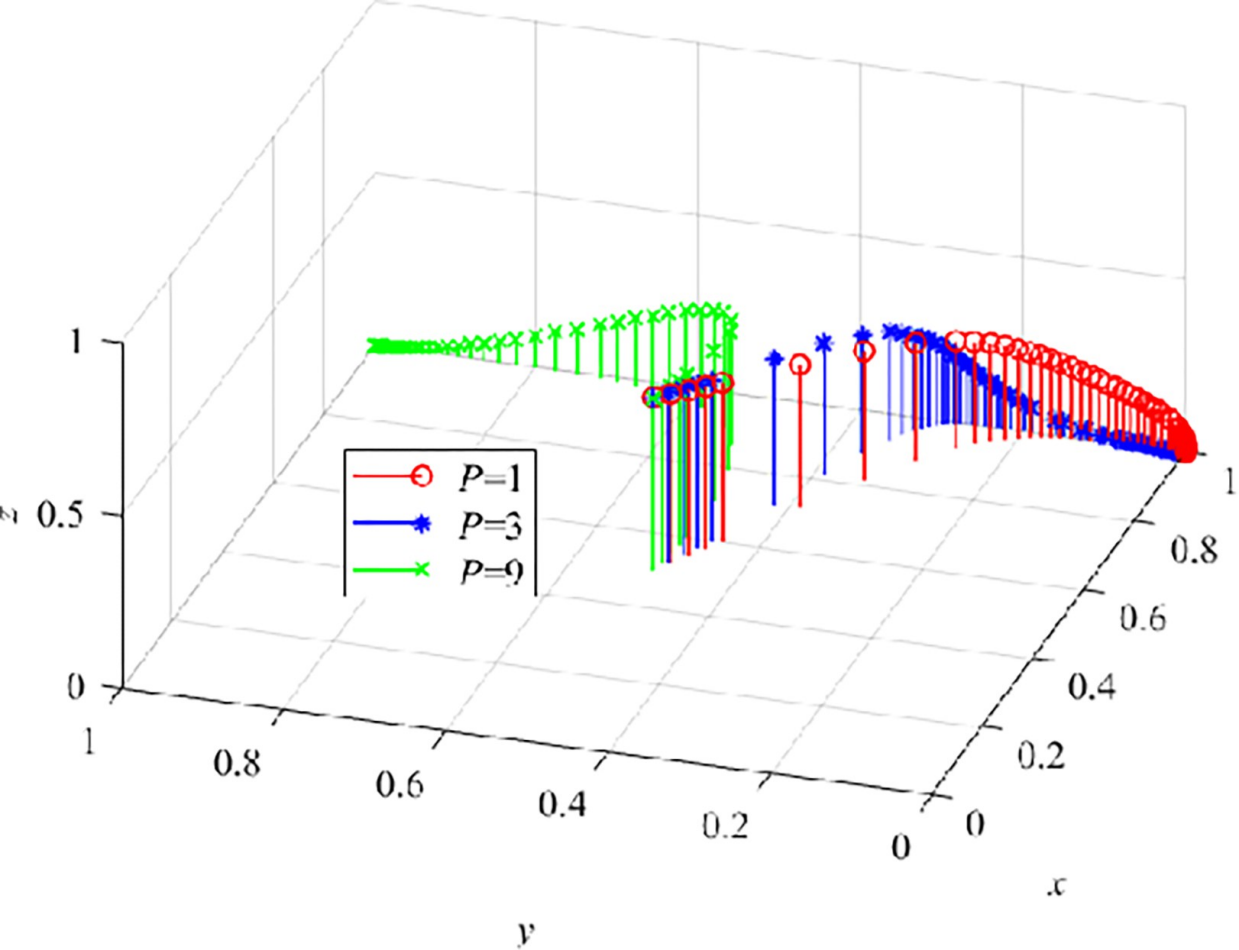
The effect of P on the dynamic evolution trend of the three-parties.

We set P as 1, 3, and 9 respectively to conduct numerical simulation of the three-party evolutionary game model. The results are shown in (Fig 12). When P = 1 and P = 3, y and z continuously decrease to 0, indicating that the lower penalty will not have an impact on CEs that still take TT, or their benefit is higher than the penalty paid. If the government increases the punishment, CEs choose to adopt VRTT when P increases to 9, indicating that the government effectively promotes CEs to adopt VRTT when VRTT enters the field of architecture.

**Fig 13:**
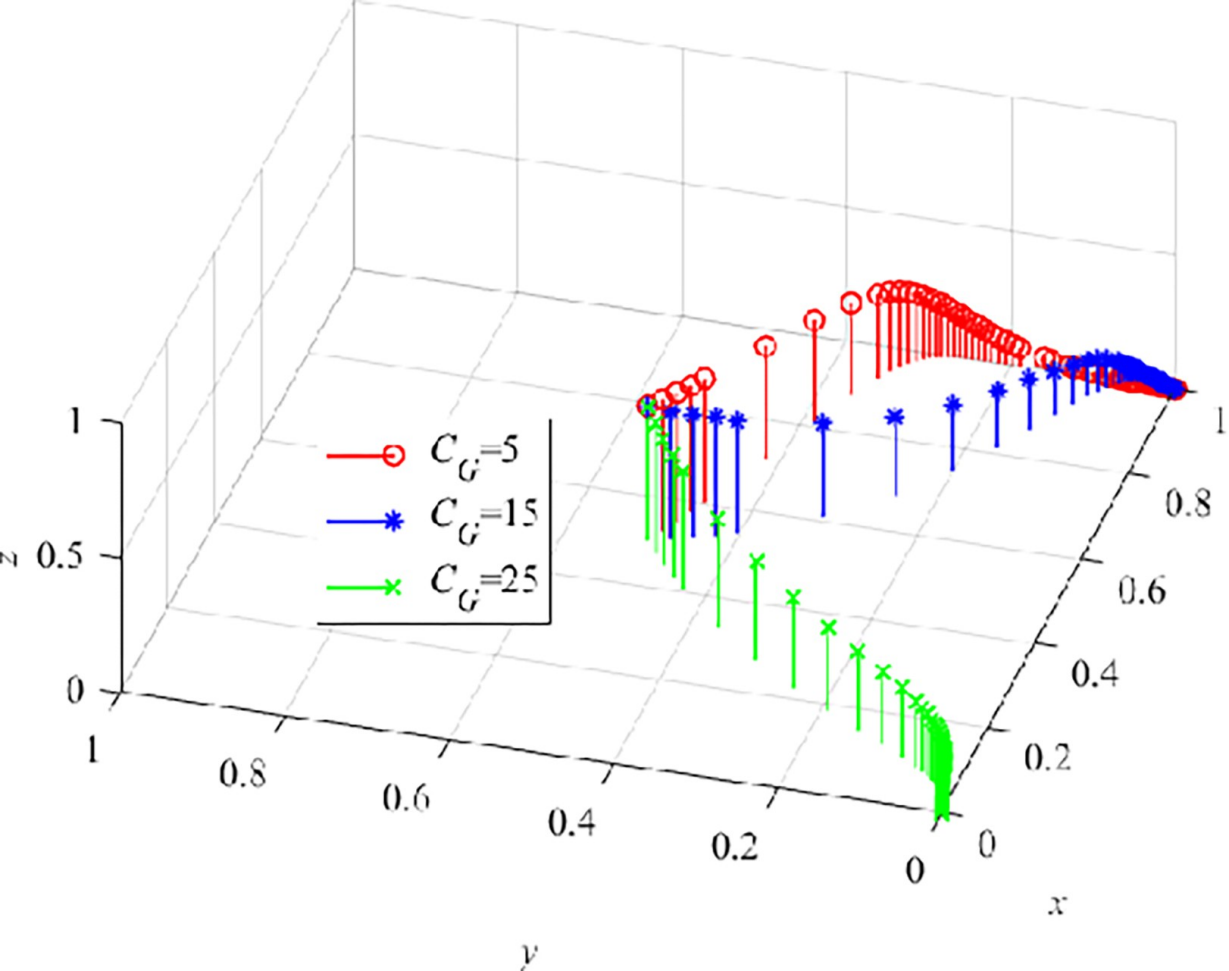
The effect of C_G_ on the dynamic evolution trend of the three-parties.

Finally, we set the cost C_G_ of the government’s active strategy selection as 5, 15, and 25 respectively, and carry out numerical simulation on the three-party evolutionary game model. The results are shown in (Fig 13). When C_G_ = 25, x decreases to 0 continuously, indicating that the government will not always pay a higher cost during the PLC. When the behavioral strategy of the three stakeholders gradually evolve to the optimal stable point, the government will withdraw from the process of VRTT entering the construction field. At this time, CEs realize that VRTT can reduce unsafe behaviors of CWs, thus reducing the occurrence of safety production accidents in enterprises [53]. Meanwhile, CWs also realize that this new training method can improve the learning efficiency compared with TT.

## Conclusions and policy implications

In this study, a three-party evolutionary game model is developed to investigate the dynamic evolutionary trends and strategic adjustment mechanisms of government, CEs and CWs. Accordingly, a series of numerical simulations are conducted to predict the evolutionary process under different stages of PLC and the influence of each factor on the dynamic system. The results of the study not only provide theoretical guidance for realizing the autonomous transformation of VRTT in the construction sector, but also provide long-term strategic insights for building a more effective and sustainable VRTT market.

There are new findings mainly in the following two aspects. Firstly, the development of VRTT in China’s construction sector is in line with the PLC. In the introduction stage of VRTT, the government initiates the market through subsidies and penalties. As CEs develop the growth stage and realize the importance of VRTT, they gradually shift to the maturity stage of not relying on the government’s intervention but relying on the autonomy-led mechanism, which leads to the participation of more and more CWs in VRTT, and provides the policy needed for the development of VRTT in China. Secondly, government fines and subsidies change the behavioral patterns of CEs and CWs, which is the system tends to the ideal stable evolutionary state. Low subsidies and penalties also hinder the development of optimal tripartite solutions. With long-term government regulation, VRTT will eventually be adopted by CEs and accepted by CWs. This will improve the learning efficiency of CWs and reduce unsafe behaviors, thus improving their own safety benefit, reducing the accident rate of CWs, and ultimately promoting the healthy development of the construction industry. Under the constraints and supervision of the government, it will maximize the interests of all parties, promote the interactive relationship among all participants, and also open up the market for VRTT in the construction industry.

In order to accelerate the application of VRTT in the construction field, this paper puts forward several suggestions.

First, the government should put in place a system that combines monetary incentives and reputational benefit to promote VRTT. It ought to give these CEs some assistance in overcoming the difficulties. To increase interests in VRTT during the introduction stage, local government can regularly host online and offline communication forums, as well as visit CEs that adopt VRTT.

Secondly, Chinese government should also encourage VRTT companies to innovate and develop in science and technology, constantly update the 3D data of their actual environment, further study mechanics and tactile sensing devices, and improve the tracking accuracy and tracking range of VR equipment. At the same time, it also encourages the application of VRTT in the medical field, military field, aerospace and other fields. This will save space and time and lead to a more efficient, self-directed learning process.

Finally, in order to reduce the financial pressure of the government, VR-related bond products can be designed to raise incentive funds for VRTT from the whole society, so as to create new financing channels for VRTT to be put into operation in the construction field. It can be piloted in China, such as Shenzhen, Hangzhou and other cities, and can be promoted nationwide after achieving certain results.

VRTT replacing TT has an important impact on improving safety production in the construction field. With the goal of promoting VRTT, it is an important way to realize the transformation on safety training in the construction field and explore the path of multi-agent system to achieve the ideal equilibrium state in the process of safety training transformation. The EGT model is used in this study to address this issue. The model’s ability to include the three facets of the government, CEs, and CWs, together with stable strategy selections, participatory decision-making, and appropriate outcomes, is very intriguing. This study offers compelling evidence that outside forces encouraging CEs to VRTT is used instead of TT. Under certain conditions, the decision of the government as the regulator can effectively regulate the evolution path. In addition, it is worth noting that with the decrease of unsafe behaviors of CWs receiving VRTT, the accident rate of CEs is reduced, indicating that CWs also plays a positive role in promoting TT transformation. These findings provide a new perspective on the dynamic evolutionary path of VRTT into the construction field, explain how the government can maximize the promotion of CEs and CWs as participants in the selection or acceptance of VRTT, and make some important policies and recommendations.

However, there are still some limitations of this study that can guide future research:(1) This study may relate to the local characteristics of the Chinese government’s governance mechanisms and is not applicable to other countries. In future studies based on other countries that are institutionally or culturally different from China, the role of the government should be specified to justify the modeling assumptions. (2) The numerical simulation in this paper is an ideal state of the assumption, and in future research, the CEs of VRTT can be adopted according to the actual to assign objective values to the model parameters to obtain accurate simulation evidence. (3) The tripartite model proposed in this paper includes only the government, CEs, and CWs. Future research could consider training companies to expand the understanding of the supply side of this booming market.

## References

[1] Kines P, Andersen LPS, Spangenberg S, Mikkelsen KL, Dyreborg J, Zohar D. Improving construction site safety through leader-based verbal safety communication. Journal of Safety Research. 2010;41(5):399–406. doi: 10.1016/j.jsr.2010.06.005 21059457

[2] Li Z. 2022-09-18. Available from: https://zhuanlan.zhihu.com/p/562479748.

[3] Shin M, Lee H-S, Park M, Moon M, Han S. A system dynamics approach for modeling construction workers’ safety attitudes and behaviors. Accident Analysis & Prevention. 2014;68:95–105. doi: 10.1016/j.aap.2013.09.019 24268437

[4] Lingard H, Rowlinson S. Behavior-based safety management in Hong Kong’s construction industry. Journal of Safety Research. 1997;28(4):243–56. 10.1016/S0022-4375(97)00010-8.

[5] Ensafi M, Thabet W, Devito S, Lewis A. Field Testing of Mixed Reality (MR) Technologies for Quality Control of As-Built Models at Project Handover: A Case Study. 2021.

[6] Rehman Khan SA, Ahmad Z, Sheikh AA, Yu Z. Digital transformation, smart technologies, and eco-innovation are paving the way toward sustainable supply chain performance. Science Progress. 2022;105(4):00368504221145648. doi: 10.1177/00368504221145648 36573795 PMC10364947

[7] Burke M, Sarpy S, Smith-Crowe K, Chan-Serafin S, Salvador R, Islam G. Relative Effectiveness of Worker Safety and Health Training Methods. American Journal of Public Health. 2006;96:315–24. doi: 10.2105/AJPH.2004.059840 16380566 PMC1470479

[8] Zhang M, Shu L, Luo X, Yuan M, Zheng X. Virtual reality technology in construction safety training: Extended technology acceptance model. Automation in Construction. 2022;135:104113. 10.1016/j.autcon.2021.104113.

[9] Zhao Z-Y, Zhao X-J, Davidson K, Zuo J. A corporate social responsibility indicator system for construction enterprises. Journal of Cleaner Production. 2012;29–30:277–89. 10.1016/j.jclepro.2011.12.036.

[10] Khan SAR, Tabish M, Yu Z. Investigating recycling decisions of internet recyclers: A step towards zero waste economy. Journal of Environmental Management. 2023;340:117968. doi: 10.1016/j.jenvman.2023.117968 37121001

[11] Okuguchi K, Yamazaki T. Global stability of unique Nash equilibrium in Cournot oligopoly and rent-seeking game. Journal of Economic Dynamics and Control. 2008;32(4):1204–11. 10.1016/j.jedc.2007.05.003.

[12] Babu SK, Krishna S, U R, Bhavani RR, editors. Virtual Reality Learning Environments for Vocational Education: A Comparison Study with Conventional Instructional Media on Knowledge Retention. 2018 IEEE 18th International Conference on Advanced Learning Technologies (ICALT); 2018 9–13 July 2018.

[13] B B, Sheshadri S, Radhakrishnan U. Vocational education technology: Rural India 2010. 21 p.

[14] Bliss J, Tidwell P, Guest M. The Effectiveness of Virtual Reality for Administering Spatial Navigation Training to Firefighters. Presence. 1997;6:73–86. doi: 10.1177/154193129503901412

[15] Karvouniari A, Michalos G, Dimitropoulos N, Makris S. An approach for exoskeleton integration in manufacturing lines using Virtual Reality techniques. Procedia CIRP. 2018;78:103–8. 10.1016/j.procir.2018.08.315.

[16] Matsas E, Vosniakos G-C. Design of a virtual reality training system for human–robot collaboration in manufacturing tasks. International Journal on Interactive Design and Manufacturing (IJIDeM). 2017;11(2):139–53. doi: 10.1007/s12008-015-0259-2

[17] Roldán JJ, Crespo E, Martín-Barrio A, Peña-Tapia E, Barrientos A. A training system for Industry 4.0 operators in complex assemblies based on virtual reality and process mining. Robotics and Computer-Integrated Manufacturing. 2019;59:305–16. 10.1016/j.rcim.2019.05.004.

[18] Oberhauser M, Dreyer D. A virtual reality flight simulator for human factors engineering. Cognition, Technology & Work. 2017;19(2):263–77. doi: 10.1007/s10111-017-0421-7

[19] Mehrfard A, Fotouhi J, Forster T, Taylor G, Fer D, Nagle D, et al. On the effectiveness of virtual reality-based training for surgical robot setup. Computer Methods in Biomechanics and Biomedical Engineering: Imaging & Visualization. 2021;9(3):243–52. doi: 10.1080/21681163.2020.1835558

[20] Scott SI, Dalsgaard T, Jepsen JV, von Buchwald C, Andersen SAW. Design and validation of a cross-specialty simulation-based training course in basic robotic surgical skills. Int J Med Robot. 2020;16(5):1–10. Epub 2020/07/29. doi: 10.1002/rcs.2138 . 32721072

[21] Lucas J, Thabet W, Worlikar P. A VR-based training program for conveyor belt safety. journal of information technology in construction. 2008;(25).

[22] Available from: https://zhuanlan.zhihu.com/p/49531013.

[23] Xu S, Sun M, Fang W, Chen K, Luo H, Zou PXW. A Bayesian-based knowledge tracing model for improving safety training outcomes in construction: An adaptive learning framework. Developments in the Built Environment. 2023;13:100111. 10.1016/j.dibe.2022.100111.

[24] Sawacha E, Naoum S, Fong D. Factors affecting safety performance on construction sites. International Journal of Project Management. 1999;17(5):309–15. 10.1016/S0263-7863(98)00042-8.

[25] Demirkesen S, Arditi D. Construction safety personnel’s perceptions of safety training practices. International Journal of Project Management. 2015;33(5):1160–9. 10.1016/j.ijproman.2015.01.007.

[26] Burke MJ, Sarpy SA, Smith-Crowe K, Chan-Serafin S, Salvador RO, Islam G. Relative effectiveness of worker safety and health training methods. Am J Public Health. 2006;96(2):315–24. Epub 2005/12/29. doi: 10.2105/AJPH.2004.059840 ; PubMed Central PMCID: PMC1470479. 16380566 PMC1470479

[27] Sacks R, Whyte J, Swissa D, Raviv G, Zhou W, Shapira A. Safety by design: dialogues between designers and builders using virtual reality. Construction Management and Economics. 2015;33:55–72. doi: 10.1080/01446193.2015.1029504

[28] Sacks R, Perlman A, Barak R. Construction safety training using immersive virtual reality. Construction Management and Economics. 2013;31:1005–17. doi: 10.1080/01446193.2013.828844

[29] Li H, Chan G, Skitmore M. Multiuser Virtual Safety Training System for Tower Crane Dismantlement. Journal of Computing in Civil Engineering. 2012;26(5):638–47. doi: 10.1061/(ASCE)CP.1943-5487.0000170

[30] Hou L, Chi H-L, Tarng W, Chai J, Panuwatwanich K, Wang X. A framework of innovative learning for skill development in complex operational tasks. Automation in Construction. 2017;83:29–40. doi: 10.1016/j.autcon.2017.07.001

[31] Eiris R, Gheisari M, Esmaeili B. Desktop-based safety training using 360-degree panorama and static virtual reality techniques: A comparative experimental study. Automation in Construction. 2020;109:102969. doi: 10.1016/j.autcon.2019.102969

[32] Tichon J, Burgess-Limerick RJ, editors. A Review of Virtual Reality as a Medium for Safety Related Training in Mining 2011.

[33] Jeelani I, Han K, Albert A. Development of virtual reality and stereo-panoramic environments for construction safety training. Engineering, Construction and Architectural Management. 2020;27(8):1853–76. doi: 10.1108/ECAM-07-2019-0391

[34] 2023.4.14. Available from: http://news.sohu.com/a/666720404_121613489.

[35] 2022.3.24. Available from: https://baijiahao.baidu.com/s?id=1728140344990245076&wfr=spider&for=pc.

[36] Ji S-f, Zhao D, Luo R-j. Evolutionary game analysis on local governments and manufacturers’ behavioral strategies: Impact of phasing out subsidies for new energy vehicles. Energy. 2019;189:116064. 10.1016/j.energy.2019.116064.

[37] Dou Y, Sun X, Ji A, Wang Y, Xue X. Development strategy for prefabricated construction projects: a tripartite evolutionary game based on prospect theory. Engineering, Construction and Architectural Management. 2021;ahead-of-print. doi: 10.1108/ECAM-05-2021-0455

[38] Coninx K, Deconinck G, Holvoet T. Who gets my flex? An evolutionary game theory analysis of flexibility market dynamics. Applied Energy. 2018;218:104–13. 10.1016/j.apenergy.2018.02.098.

[39] Eissa R, Eid M, Elbeltagi E. Current Applications of Game Theory in Construction Engineering and Management Research: A Social Network Analysis Approach. Journal of Construction Engineering and Management. 2021;147. doi: 10.1061/(ASCE)CO.1943-7862.0002085

[40] Chen Z, Wang T. Photovoltaic subsidy withdrawal: An evolutionary game analysis of the impact on Chinese stakeholders’ strategic choices. Solar Energy. 2022;241:302–14. doi: 10.1016/j.solener.2022.04.054

[41] A CC, B DP, B MS. A game theory-based assessment of the implementation of green building in Israel—ScienceDirect. Building and Environment. 2017;125:122–8.

[42] Feng T, Tai S, Sun C, Man Q. Study on Cooperative Mechanism of Prefabricated Producers Based on Evolutionary Game Theory. Mathematical Problems in Engineering. 2017;2017:1676045. doi: 10.1155/2017/1676045

[43] Jing S, Zhang Z, Yan J. Government Supervision Mode Selection in the Promotion Period of Prefabricated Construction Using Evolutionary Game. Mathematical Problems in Engineering. 2020;2020:7105617. doi: 10.1155/2020/7105617

[44] Lv J, Lin M, Zhou W, Xu M. How PPP Renegotiation Behaviors Evolve with Traffic Changes: Evolutionary Game Approach. Journal of Construction Engineering and Management. 2021;147(5):04021032. doi: 10.1061/(ASCE)CO.1943-7862.0002024

[45] Tuyls K, Parsons S. What evolutionary game theory tells us about multiagent learning. Artificial Intelligence. 2007;171(7):406–16. doi: 10.1016/j.artint.2007.01.004

[46] Zhang L, Chen L, Wu Z, Sizhen Z, Song H. Investigating Young Consumers’ Purchasing Intention of Green Housing in China. Sustainability. 2018;10:1044. doi: 10.3390/su10041044

[47] Zhao H, Liu X, Wang Y. Evolutionary game analysis of opportunistic behavior of Sponge City PPP projects: a perceived value perspective. Scientific Reports. 2022;12(1):8798. doi: 10.1038/s41598-022-12830-0 35614166 PMC9132952

[48] Fawcett T, Hamblin S, Giraldeau L-A. Exposing the behavioral gambit: The evolution of learning and decision rules. Behavioral Ecology. 2012;24:2–11. doi: 10.1093/beheco/ars085

[49] Lv J, Lin M, Zhou W, Xu M. How PPP Renegotiation Behaviors Evolve with Traffic Changes: Evolutionary Game Approach. Journal of Construction Engineering and Management. 2021;147:04021032. doi: 10.1061/(ASCE)CO.1943-7862.0002024

[50] Jiang S, Wei X, Jia J, Ma G. Toward sustaining the development of green residential buildings in China: A tripartite evolutionary game analysis. Building and Environment. 2022;223:109466. doi: 10.1016/j.buildenv.2022.109466

[51] Yinnan HE, Ruxiang QIN. Autonomous rectification behavior of coal mine safety hazards under a gambling mind: From an evolutionary game perspective. Process Safety and Environmental Protection. 2023;169:840–9. doi: 10.1016/j.psep.2022.11.064

[52] Raymond V. International investment and international trade in the product cycle. Quarterly Journal of Economics. 1966;(2):2.

[53] Zhang W, Zhu S, Zhang X, Zhao T. Identification of critical causes of construction accidents in China using a model based on system thinking and case analysis. Safety Science. 2020;121:606–18. doi: 10.1016/j.ssci.2019.04.038

[54] Luo LZ, Chao M, Shen LY, Li ZD. Risk factors affecting practitioners’ attitudes toward the implementation of an industrialized building system. Engineering Construction & Architectural Management. 2015;22(6):622–43.

[55] Lyapunov AM. The general problem of the stability of motion. International Journal of Control. 1992;55(3):531–4. doi: 10.1080/00207179208934253

[56] Friedman D. Evolutionary Games in Economics. Econometrica. 1991;59:637–66. doi: 10.2307/2938222

[57] Wainwright J. A dynamical systems approach to Bianchi cosmologies: orthogonal models of class A. Classical and Quantum Gravity. 1989;6(10):1409. doi: 10.1088/0264-9381/6/10/011

[58] Su Y. Multi-agent evolutionary game in the recycling utilization of construction waste. Science of The Total Environment. 2020;738:139826. doi: 10.1016/j.scitotenv.2020.139826 32562906

[59] Xu Z, Zheng N. Incorporating Virtual Reality Technology in Safety Training Solution for Construction Site of Urban Cities. Sustainability. 2020;13:243. doi: 10.3390/su13010243

